# Emergent Stratification in Solid Tumors Selects for Reduced Cohesion of Tumor Cells: A Multi-Cell, Virtual-Tissue Model of Tumor Evolution Using CompuCell3D

**DOI:** 10.1371/journal.pone.0127972

**Published:** 2015-06-17

**Authors:** Maciej H. Swat, Gilberto L. Thomas, Abbas Shirinifard, Sherry G. Clendenon, James A. Glazier

**Affiliations:** 1 Biocomplexity Institute and Department of Physics, Indiana University, Bloomington, Indiana, USA; 2 Instituto de Física, Universidade Federal do Rio Grande do Sul, Porto Alegre, Brazil; 3 St. Jude Children’s Research Hospital, Department of Information Sciences, Division of Clinical Informatics, Memphis, USA; George Washington University, UNITED STATES

## Abstract

Tumor cells and structure both evolve due to heritable variation of cell behaviors and selection over periods of weeks to years (*somatic evolution*). Micro-environmental factors exert selection pressures on tumor-cell behaviors, which influence both the rate and direction of evolution of specific behaviors, especially the development of tumor-cell aggression and resistance to chemotherapies. In this paper, we present, step-by-step, the development of a multi-cell, virtual-tissue model of tumor somatic evolution, simulated using the open-source CompuCell3D modeling environment. Our model includes essential cell behaviors, microenvironmental components and their interactions. Our model provides a platform for exploring selection pressures leading to the evolution of tumor-cell aggression, showing that emergent stratification into regions with different cell survival rates drives the evolution of less cohesive cells with lower levels of cadherins and higher levels of integrins. Such reduced cohesivity is a key hallmark in the progression of many types of solid tumors.

## Introduction

Tumor cells and tumor structure both evolve over periods of weeks to years (*somatic evolution*). Microenvironmental factors such as levels of nutrients and oxygen, growth factors, host immune response and the structure and composition of the extracellular matrix (*ECM*), exert selection pressures on tumor cells, which influence both the rate and direction of evolution of specific behaviors (often designated *hallmarks*), especially the development of tumor-cell aggression and resistance to chemotherapies [1]. A particular problem for cancer treatment is that recurrent tumors are typically more aggressive than primary tumors, and that tumors often become resistant to chemotherapeutic agents. Thus treatment may have the iatrogenic effect of making the cancer more aggressive and less treatable. Cells within a single tumor vary in their morphological and molecular features. The tumor microenvironment is also heterogeneous, with marked spatial and temporal variations in the concentrations of metabolites and therapeutics, confounding efforts to assess how specific selection pressures favor particular cell phenotypes *in vivo*.

Somatic evolution can also lead to apparently paradoxical responses to treatment. *E.g.*, metronomic chemotherapy, the near continuous administration of cytotoxic drugs at low doses with no extended interruption, induces dormancy in some types of cancers [2], though the effectiveness of metronomic therapy is greater in chemo-naïve than in previously-treated patients. Nutrient starvation (*e.g.* due to antiangiogenics) can cause tumor cells to shrink and enter a state of reversible dormancy, resuming active growth and proliferation when the microenvironment changes and more nutrients become available [3]. Starvation often (but not always) reduces the effectiveness of chemotherapies. Thus the same treatment may help some patients with a given tumor type and harm others. Despite the growing number of available tests for specific markers in tumors, in many cases, we cannot predict which patients a treatment regime will benefit and which it will harm. Understanding the interacting evolutionary pressures within a tumor will therefore be an essential step in enabling personalized and more effective treatment regimes. Because resources are limited and the number of potential treatment regimes limitless, exhaustive combinatoric patient-based trials with different combinations and regimes of drugs range from impractical to impossible. In addition, such studies can only determine optimal conditions for population-average responses and not for personalized treatment of individuals. Ideally, we would like to be able to predict how a tumor in a specific patient will react to a given treatment regime based on easily measured biomarkers. Virtual-tissue models of tumors may provide a pathway to developing such predictions.

Hybrid virtual-tissue models of tumor growth (e.g. [4] and review in [5]) are mathematical frameworks which can capture the complex interactions of tumor growth with intercellular and intracellular signaling across the multiple scales modulating cancer progression. The Glazier-Graner-Hogeweg (**GGH**) model [6] is a multi-cell hybrid virtual-tissue model that implements cell behaviors and interactions to predict tissue-scale dynamics. GGH model applications include embryonic development and development-related diseases, including angiogenesis [7–10], choroidal neovascularization in the retina [11], avascular [12] and vascular [7] tumor growth, chick-limb growth [13] and somitogenesis [14].

CompuCell3D (*CC3D*) [15] is a free, fully open-source modeling environment for developing and running GGH-based multi-cell, multi-scale virtual-tissue simulations. CC3D provides many multi-cell virtual-tissue model components, including GGH solvers for cell movement, PDE solvers for chemical fields, reaction-kinetics solvers for biochemical networks, visualization tools, *etc*. CC3D supports compact model specification using a combination of CompuCell3D Markup Language(*CC3DML*) and Python scripting. CC3D allows specification of arbitrary subcellular signaling, regulatory and metabolic dynamic network models using SBML and executes these models and their control of cell-level and tissue-level behaviors. CC3D also supports large-scale/macroscopic continuum models via Python scripting. CompuCell3D users can easily share models, which stimulates simulation code reuse and facilitates collaborative model development. CC3D’s use of CC3DML and Python for model specification greatly reduces the effort to develop biological simulations compared to traditional simulation development using C++ or Fortran. The CompuCell3D website (www.compucell3d.org) provides free downloads of the CompuCell3D environment for the Windows, OSX and Linux operating systems and all model code.

This paper demonstrates step-by-step, the creation and execution of a multi-cell virtual-tissue computational model of tumor growth and evolution, providing a platform for the study of the influence of micro-environmental factors on tumor progression. The Appendix provides listings of all models in this paper. Our model focuses on small, early-stage benign tumors and on early development of metastases rather than on developed primary tumors, to identify the pattern of cell-behavior selection. While the average number of cells in our 2D model is less than 1000 at any time and the total cell turnover is on the order of tens of thousands of cells, much fewer than in even a small real tumor, we can map these simplified model tumors onto real tumors either by considering each model cell to represent an ensemble of hundreds or thousands of real cells or by considering each model tumor to represent a peripheral micro-portion of a much larger tumor mass.

## Methods: Multi-Cell Virtual-Tissue Models of Tumor Evolution

Our model includes multiple cell agents of different types. Each cell agent has a set of defining properties, including its volume, surface area, intrinsic motility and two distinct classes of adhesion molecules on its membrane. Each cell interacts with neighboring cells and surrounding ECM and stromal tissue primarily by adhesion. We model ECM and stromal tissue as a simple uniform medium, rather than representing individual ECM fibers and stromal cells explicitly and model cells’ adhesion to ECM fibers and stromal cells using appropriate contact-energy coefficients—see later sections. We also include a diffusive growth-limiting nutrient (*glucose*). In our model, sufficient glucose availability promotes cell growth and proliferation, while glucose depletion causes accumulating cell damage, which may lead to cell death. We assume that all cells except *stem-like* cancer cells can undergo a limited number of cell cycles (*Senescence*). We represent the effects of all heritable genetic and epigenetic mutations as random variations of the parameters controlling cell behaviors, with variation occurring immediately after a cell division.

Outline: We first describe briefly model components (*i.e*., objects and processes) and their biological relevance and background. We then discuss the simplifying assumptions that allow us to abstract solid-tumor biology to construct a biomodel. Appendix 1 provides an implementation of this biomodel as a simulation, specified in CC3DML and Python. Finally, we compare the simulation results to experiments to estimate the model’s parameters.

### Biological Components

**Cells**

Biomodel: Our generic solid tumor includes two main classes of generalized cells: *tumor cells* and *stromal tissue*. Tumor cells have two subtypes: *stem-like cancer cells* and *somatic cancer cells*. All tumor cells assume one of three possible states: *Proliferating* cancer cells—PCancer (PC), PStem (PS), which grow and divide, *quiescent* cancer cells—QCancer (QC), QStem (QS), which do not grow, and Necrotic cells (N), which die. An extracellular Medium (M) represents an aggregate of stromal cells and extracellular matrix (*ECM*).

We define a separate CC3D *cell type* for each class of cells which has a distinct set of biological behaviors and properties. While all cells of a given type have the same initial list of defining parameters, the properties of each cell of a given type can change during a simulation. We usually limit the number of cell types to no more than 15 to make the model intelligible (For our specific CC3D implementation of cell types, see Table 2).

**Fields**

Biomodel: Tumor growth *in vivo* depends on the levels of multiple diffusing substances, including blood nutrients (*e.g.* glucose and fatty acids), tissue oxygen, growth factors and pH. In our model, we assume that glucose is the main growth-limiting nutrient and include a diffusing field (*G*) representing glucose (see “Glucose Transport” section for details).

### Biological Processes

**Cell Motility, Time and the Glazier-Graner-Hogeweg Model**

Biomodel: Cells move by making cytoskeletally-driven membrane protrusions and retractions and by making and breaking connections with their neighbors and the ECM. In the absence of external stimuli (*e.g.*, gradients of chemical attractants or substrate properties), cells like fibroblasts migrate in a persistent random-walk pattern, where the cell typically moves at least one cell diameter in a given direction before changing direction. In our model, we assume that tumor cells in a cluster migrate via a random walk without a prespecified persistence length.

To simulate the dynamic motility of cells, we use the GGH model, also known as the Cellular Potts Model (**CPM**), a multi-cell, lattice-based, stochastic methodology for representing tissues. The GGH model uses spatially-extended domains of voxels on a fixed *cell lattice* to represent cells. Since such domains may also represent cell subcomponents, clusters of cells or portions of ECM, we call the domains *generalized cells*. Each voxel in the cell lattice has a position $\vec{x}$ and an *index* *σ* ($\vec{x}$) denoting the index of the generalized cell to which it belongs. For convenience, we also assign a *cell type* *τ* (*σ*) to each generalized cell *σ*. One or more generalized cells can represent a single biological cell. In the latter case, the biological cell corresponds to a cluster of generalized *subcells*, which we can use to represent cell compartments or cell polarity, or to assemble cells with complex shapes.

To model the dynamics of generalized cells, we associate an *effective energy* term with each generalized-cell behavior which involves motion (*e.g.*, chemotaxis), force-mediated interaction (*e.g.*, cell-cell adhesion, an external force), or size or shape constraint (*e.g.*, the cell volume), *etc*.… In some cases, an effective energy term represents a physical interaction energy, *e.g.*, cell-cell adhesion. In other cases, (*e.g.*, chemotaxis) the effective energy is a shorthand to produce the desired behavior of cells and does not correspond to an actual energy.

To model a motile cell that has a defined volume and surface area, we use two effective energy terms in the form of constraints: the *cell-volume* (first term) and *cell-surface constraints* (second term):

$$\begin{array}{c} H=\sum_{\sigma }\lambda_{{}_{\sigma }}^{vol}{\left(v_{\sigma }-V_{\sigma }\right)}^{2}+\sum_{\sigma }\lambda_{{}_{\sigma }}^{sur}{\left(s_{\sigma }-S_{\sigma }\right)}^{2}, \end{array}$$ (1)

where *v*_*σ*_ and *s*_*σ*_ denote a generalized-cell’s instantaneous volume or instantaneous surface area and *V*_*σ*_ and *S*_*σ*_ denote a generalized-cell’s target volume and target surface area, respectively. The constraints are quadratic and vanish when *v*_*σ*_ = *V*_*σ*_ and *s*_*σ*_ = *S*_*σ*_. $\lambda_{\sigma }^{vol}$ and $\lambda_{\sigma }^{sur}$ are the constraint *strengths* which correspond to elastic moduli (the higher $\lambda_{\sigma }^{vol}$ or $\lambda_{\sigma }^{sur}$ the more energy a given deviation from the target volume or surface area costs).

The GGH model represents cytoskeletally-driven cell motility as a series of stochastic voxel-copy attempts. For each attempt, we randomly select a *target site*, $\vec{i}$, in the cell lattice, and a neighboring *source site* $\vec{j}$. If different generalized cells occupy these two sites, we calculate change in the energy (Δ*H*) that would occur if we were to copy the index in the the source-site voxel onto the target-site voxel. For almost all cell behaviors and interactions, the evaluation of Δ*H* requires calculations localized to the vicinity of the target voxel only.

The probability of accepting a voxel-copy attempt (*i.e*., overwriting the value in the target-site voxel) is:

$$\begin{array}{c} P(\sigma_{\vec{i}}\to \sigma_{\vec{j}})=\{\begin{array}{cc} 1, & \text{for}\;\;\Delta H\leq 0\\ f(\Delta H), & \text{for}\;\;\Delta H>0, \end{array} \end{array}$$ (2)

where *f* (Δ*H*) is a decreasing function, bounded between 0 and 1. Here as in most GGH models, we set

$$\begin{array}{c} f(\Delta H)=e^{-\frac{\Delta H}{T_{m}}} \end{array}$$ (3)

where *T*_*m*_ is a parameter describing the amplitude of cell-membrane fluctuations. *T*_*m*_ can be a global parameter, cell specific or cell-type specific.

The net effect of the GGH voxel-copy algorithm is to lower the effective energy of the generalized-cell configuration in a manner consistent with the biologically-relevant “guidelines” in the effective energy: cells maintain volumes close to their target values, mutually-adhesive cells stick together, mutually repulsive cells separate, *etc*.…. The ability to make voxel copies which temporarily increase the effective energy helps avoid trapping the configuration in local effective-energy minima. The algorithm evolves the cell-lattice configuration to simultaneously satisfy the constraints, to the extent to which they are compatible. Generalized-cell movements are perfectly damped (*i.e*., average velocities are proportional to applied forces), which makes the updating method numerically stable, making the GGH model a robust tool for building virtual-tissue models [16].

The average value of the ratio $\frac{\text{Δ}\;H}{T_{m}}$ for a given generalized cell determines the amplitude of fluctuations of the generalized-cell’s boundaries. High $\frac{\text{Δ}\;H}{T_{m}}$ results in rigid, barely- or non-motile generalized cells and little cell rearrangement. For low $\frac{\text{Δ}\;H}{T_{m}}$, large fluctuations allow a high degree of generalized-cell motility and rearrangement. For extremely low $\frac{\text{Δ}\;H}{T_{m}}$, generalized cells may fragment in the absence of a constraint sufficient to maintain the integrity of the borders between them. Because $\frac{\text{Δ}\;H}{T_{m}}$ is a ratio, we can achieve appropriate generalized-cell motility by varying either *T*_*m*_ or Δ*H*. Varying *T*_*m*_ allows us to explore the impact of global changes in cytoskeletal activity. Varying Δ*H* allows us to control the relative motility of the cell types or of individual generalized cells by varying, for example, $\lambda_{\sigma }^{vol}$, $\lambda_{\sigma }^{sur}$, *V*_*σ*_, or *S*_*σ*_.

The generalized cell representing the stromal material around the tumor has unconstrained volume and surface area. Medium voxels can be both source voxels, *e.g.*, during retraction of the trailing-edge of a generalized cell, and target voxels, *e.g.* during formation of lamellipodia. Since Medium represents largely passive material, We use the amplitude of cytoskeletal fluctuations of the non-Medium target or source generalized cell to determine the acceptance probability for a voxel-copy involving Medium.

GGH simulations measure simulation time in terms of Monte Carlo Step units (*MCS*), where MCSs are *N* voxel-copy attempts, where *N* is the number of voxels in the cell lattice, and sets the natural unit of time in the model. The conversion between MCS and experimental time depends on the average cell motility. In biologically-meaningful situations, MCSs and experimental time are proportional.

Parameter Estimation: In CC3D, the size of the cell-lattice voxel sets the spatial resolution of the simulation. Here a square cell-lattice voxel (2D) represents 16 *μm* ^2^. Our tumor cells have an initial volume of 256 *μ*m^2^. We relate the simulation’s MCS time-scale to minutes by comparing cell-migration speeds in simulations to typical cell-migration speeds in experiments. Simulated tumor cells migrate at speeds of about 0.1 voxel/MCS. Experimentally, tumor-cell migration speeds range between 2 *μ*m/hour to 12 *μ*m/hour [17]. Matching the experimental value of 4 *μ*m/hour sets one MCS to 6 min. Both [18] and [19] find that the active cells that lead to metastases move with relatively high speeds.

ECM fibers play a crucial role in enabling such rapid cell-migration. Cancer invasion models based on game theory reach similar conclusions [20], as do other CA models (*e.g.*, [21]) that focus on the relative significance of the rates of cell death, proliferation and migration on tumor growth and spread. However, none of these models captures the role of cell adhesion changes (one of the classical hallmarks of cancer progression) on the initiation of the cancer invasion. Our model concentrates on the evolution of cell adhesion in tumors due to the tumor’s changing microenvironment. While, it does not explicitly represent ECM fibers or the intracellular signaling pathways that help control cell migration [19], we hope to include these more detailed submodels in future studies.

**Cell Adhesion**

Biomodel: Cell adhesion due to molecular binding of transmembrane adhesion receptors on one cell to either ligand transmembrane adhesion receptors on another cell or to the ECM is one of the most important interactions during tissue morphogenesis and maintenance [22]. A wide variety of adhesion molecules can mediate both cell-cell and cell-ECM adhesion. Detailed models of adhesion are complex because of the multiple interactions affecting these molecules, which occur in both the cytoplasmic and extracellular domains [23, 24]. Here, we assume that a single cadherin species mediates cell-cell adhesion via homotypic cadherin-cadherin binding and a single integrin species mediates cell-stromal tissue adhesion via heterotypic integrin-fibronectin binding. We simplify the strength of adhesion as the product of the number of bonds between two adhering objects and the strength per bond. Bond formation contributes a negative energy to the total effective energy in the model, and breaking a bond requires an energy at least equal to this binding energy. represent inter-cellular adhesive interactions, we add adhesion term to the effective-energy in Eq (1):

$$\begin{array}{c} H=\sum_{\vec{x}}\sum_{\vec{y}}^{N_{\max}}J_{\sigma (\vec{x}),\sigma (\vec{y})}(1-\delta_{\sigma (\vec{x}),\sigma (\vec{y})})+\sum_{\sigma }\lambda_{{}_{\sigma }}^{vol}{\left(v_{\sigma }-V_{\sigma }\right)}^{2}+\sum_{\sigma }\lambda_{{}_{\sigma }}^{sur}{\left(s_{\sigma }-S_{\sigma }\right)}^{2}. \end{array}$$ (4)

The outer sum of the (first) adhesion term iterates over all cell-lattice voxels. We refer to the closest voxels to $\vec{x}$ as 1^*st*^ order (nearest neighbor) voxels, and voxels further away as 2^*nd*^, 3^*rd*^ order, *etc*.… neighbors of voxel $\vec{x}$. The inner sum (over $\vec{y}$) iterates over all voxels in the neighborhood of voxel $\vec{x}$ up to order *N*_max_. $J_{\sigma (\vec{x}),\sigma (\vec{y})}$ is the adhesion energy per unit contact area between two generalized cells—*σ* ($\vec{x}$) and *σ* ($\vec{y}$). *δ* is the Kronecker delta function:

$$\begin{array}{c} \delta (x,y)=\{\begin{array}{cc} 0, & \text{for}\;\;x\neq y\\ 1, & \text{for}\;\;x=y \end{array} \end{array}$$ (5)

and the $\left(1-\delta_{\sigma (\vec{x}),\sigma (\vec{y})}\right)$ factor ensures that we only count energies between voxels belonging to different cells. Inclusion of the adhesion term in Eq. (4) reduces the amount of cell-cell contact between generalized cells with high values of $J_{\sigma (\vec{x}),\sigma (\vec{y})}$ and increases the amount of contact between generalized cells with low values of $J_{\sigma (\vec{x}),\sigma (\vec{y})}$. In our model, we express $J_{\sigma (\vec{x}),\sigma (\vec{y})}$ as a function of the adhesion-molecule concentrations on generalized-cells’ surfaces. As we noted above, several classes of adhesion molecules can reside on a generalized-cell’s surface and we treat the ECM and stromal cells as a generalized cell. The binding energy per unit area between two adhering generalized cells due to integrin-like or cadherin-like adhesion molecules is:

$$\begin{array}{c} {\tilde{E}}_{m,n}^{i,j}=k_{m,n}F\;(N_{m}^{i},N_{n}^{j}), \end{array}$$ (6)

where, for cells *i* and *j*, *m* and *n* are classes of adhesion molecules, *k*_*m*, *n*_ the affinity coefficient for those species, $N_{m}^{i}$ and $N_{n}^{j}$ their densities (the number of adhesion molecules of classes *m* and *n*, respectively, per unit area), and *F* is a function which describes the phenomenological relationship between the densities and the binding energy. In our model, we set

$$\begin{array}{c} F\;(x,y)=\min(x,y), \end{array}$$ (7)

which assumes that each molecule of class *m* on cell *i* can bind only once to a molecule *n* on cell *j*.

Including all possible combinations of bonds between different classes of adhesion molecules, the adhesion energy per unit area is:

$$\begin{array}{c} {\tilde{E}}^{i,j}=\sum_{m,n}k_{m,n}\min(N_{m}^{i},N_{n}^{j}). \end{array}$$ (8)

To calculate the net contribution of adhesion to the effective energy, we sum the adhesion energies at every cell-cell and cell-stroma (cell-Medium) interface:

$$\begin{array}{c} E_{adh}=\sum_{\vec{x}}\sum_{\vec{y}}^{N_{\max}}{\tilde{E}}^{\sigma (\vec{x}),\sigma (\vec{y})}(1-\delta_{\sigma (\vec{x}),\sigma (\vec{y})}), \end{array}$$ (9)

where we have replaced indicies *i* and *j* with *σ* ($\vec{x}$) and *σ* ($\vec{y}$) respectively, following the notation of Eq (1). Comparing Eqs. (4), (8) and (9), we see that:

$$\begin{array}{c} J_{\sigma (\vec{x}),\sigma (\vec{y})}={\tilde{E}}^{\sigma (\vec{x}),\sigma (\vec{y})}=\sum_{m,n}k_{m,n}\min(N_{m}^{\sigma (\vec{x})},N_{n}^{\sigma (\vec{y})}). \end{array}$$ (10)

Using Eq (10) we can express the GGH effective energy as:

$$\begin{array}{ccc} H & = & \sum_{\vec{x}}\sum_{\vec{y}}^{N_{\max}}\sum_{m,n}k_{m,n}\min(N_{m}^{\sigma (\vec{x})},N_{n}^{\sigma (\vec{y})})(1-\delta_{\sigma (\vec{x}),\sigma (\vec{y})})\\ & & +\sum_{\sigma }\lambda_{{}_{\sigma }}^{vol}{\left(v_{\sigma }-V_{\sigma }\right)}^{2}+\sum_{\sigma }\lambda_{{}_{\sigma }}^{sur}{\left(s_{\sigma }-S_{\sigma }\right)}^{2}. \end{array}$$ (11)

See Table 3 for the CC3DML implementation of the adhesion energy and for initial parameter estimates.

Parameter Estimation: To form an initially cohesive solid tumor, we set (see Table 3) the AdhesionMoleculeDensity and the homotypic and heterotypic BindingParameters to values that produce a positive surface tension between tumor cells and Medium, which we calculate using the surface tension formula:

$$\begin{array}{ccc} \gamma & = & -\min(\text{Int},\text{FN})\times \text{BindingParameter}(\text{Int},\text{FN})\\ & & +\;\frac{\min(\text{Cad},\text{Cad})\times \text{BindingParameter}(\text{Cad},\text{Cad})}{2}. \end{array}$$ (12)

A high positive *γ* produces a cohesive cluster of tumor cells, while zero (non-cohesive cells) or negative *γ* (invasive cells) indicates that tumor cells can separate from the tumor cluster and invade the surrounding Medium. Positive surface tension corresponds to cell-lattice configurations where replacing cell-cell interfaces with cell-Medium interfaces requires energy input—hence, in the absence of external stimuli, cells stick together, while negative surface tension causes cells to tend to separate, because creating cell-medium interfaces is energetically favorable.

We assume that heterotypic adhesion is weaker than homotypic adhesion and limit both Int and Cad densities to 16 units. Higher Int and Cad densities can cause generalized cells to fragment and introduce cell-lattice-alignment artifacts (see CC3D manual for details). In our model, the initial cohesive solid tumor has a *γ* of about 2.4. Later in the simulation, some cells evolve to express lower levels of Cad and higher levels of Int, reducing the surface tension and causing the cells to invade the Medium.

**Glucose Transport**

Biomodel: The blood vessels in biologically-normal stromal tissue supply nutrients at a rate which depends on the metabolic needs of the tissue, keeping nutrient levels in stromal tissue within physiologically-normal ranges. In our model, Medium supplies *G* at a constant rate *α*. Since Medium represents the normal cells in the stromal tissue as well as the ECM, it also takes up glucose at a rate proportional to the local glucose concentration *G*(*x*):

$$\begin{array}{c} U_{G}^{\text{ECM}}=\alpha -\epsilon G\left(x\right), \end{array}$$ (13)

where *ϵ* is the first-order glucose uptake rate. In the absence of tumor cells, *G* approaches the equilibrium glucose concentration for normal stroma. Tumor cells take up glucose at a rate which is a Michaelis-Menten function of the local glucose concentration *G*(*x*), cell-type (*τ*) and cell-state (*s*):

$$\begin{array}{c} U_{G}^{tumor}(x)=-\frac{u_{\max}(\tau ,s)G(x)}{G(x)+K(\tau ,s)}, \end{array}$$ (14)

where *u*_max_(*τ*, *s*) is the maximum uptake rate and *K*(*τ*, *s*) is a Michaelis constant, both of which depend on the cell-type (*τ*) and cell-state (*s*).

Glucose diffuses at a constant rate *D*_*G*_, with time-dependent secretion and uptake:

$$\begin{array}{ccc} \frac{\partial G(x)}{\partial t} & = & D_{G}\nabla^{2}G(x)+\delta (\tau (\sigma (x)),tumor)U_{G}^{tumor}\;(x)\\ & & +\delta (\tau (\sigma (x)),\text{Medium})U_{G}^{\text{Medium}}\;(x), \end{array}$$ (15)

where,

$$\begin{array}{c} \delta (\tau (\sigma (x)),Tumor)=1\;\;\text{inside}\;\text{tumor}\;\text{cells}\;\text{(types:}\;\text{PC},\;\text{QC},\;\text{PS},\;\text{QS}) \end{array}$$ (16)

and

$$\begin{array}{c} \delta (\tau (\sigma (x)),\;\;\text{Medium})=1\text{in}\;\text{stromal}\;\text{tissue}\;(\mathrm{Medium}). \end{array}$$ (17)

CompuCell3D includes a steady-state diffusion-equation solver, which is appropriate for a fast-diffusing species like glucose. Table 4 shows how the model specifies the behavior of glucose using this solver.

Parameter Estimation: We set the Glucose diffusion coefficient to 600 *μ*m^2^/*s* which corresponds to 13500 voxel^2^/MCS. In the absence of tumor cells, Glucose concentration in the simulated stromal tissue approaches an equilibrium Glucose concentration of 5mM. 3D does not impose specific units for field concentration. We use femto-mole (*fmol*) per voxel as our unit and impose a scaling factor so that 5mM corresponds to 0.32 fmol/voxel.

Experimentally, cell glucose consumption rates are about 0.1 fmol/cell/s, which corresponds to 2.25 fmol/voxel/MCS, so we set the Glucose consumption rate of tumor cells in their queiscent phase to be 0.75 of that of tumor cells in their proliferating phase (1.69 fmol/voxel/MCS). We assume that stromal cells (represented implicitly by Medium) are the same size as tumor cells, occupy 20% of the stromal volume, and consume Glucose at $\frac{1}{3}$ the rate of tumor cells (0.75 fmol/voxel/MCS). For these parameters, Medium must supply Glucose at a rate of 0.145 fmol/voxel/MCS to maintain a stationary and uniform Glucose concentration of 0.32 fmol/voxel over the entire Medium in the absence of a tumor. If the Glucose concentration is stationary and uniform, both the left-hand side of Eq (15) and the diffusion term on the right hand side are zero, so the decay coefficient of Glucose (the first-order consumption rate), *ϵ* = 0.45. We set the Michaelis constant of Glucose (MichaelisMentenCoef) to 0.04 mM (0.00256 fmol/voxel).

**Cell-State Transitions**

Biomodel: Experimentally, microenvironmental factors including mechanical stress, hydrostatic pressure, low pH and starvation can cause temporary or permanent changes in tumor cells [25, 26]. In our model, however, we include only cell-state transitions due to nutrient availability. During a period of starvation (in a low-nutrient regime), tumor cells accumulate *damage* at rates that depend on their local nutrient levels. When accumulated damage in a cell passes a threshold which depends on the cell type and state, the cell dies (an irreversible cell-type transition). When a tumor cell divides, it resets its damage level to zero, so daughter cells do not inherit any accumulated damage from their parent.

**Fig 1:**
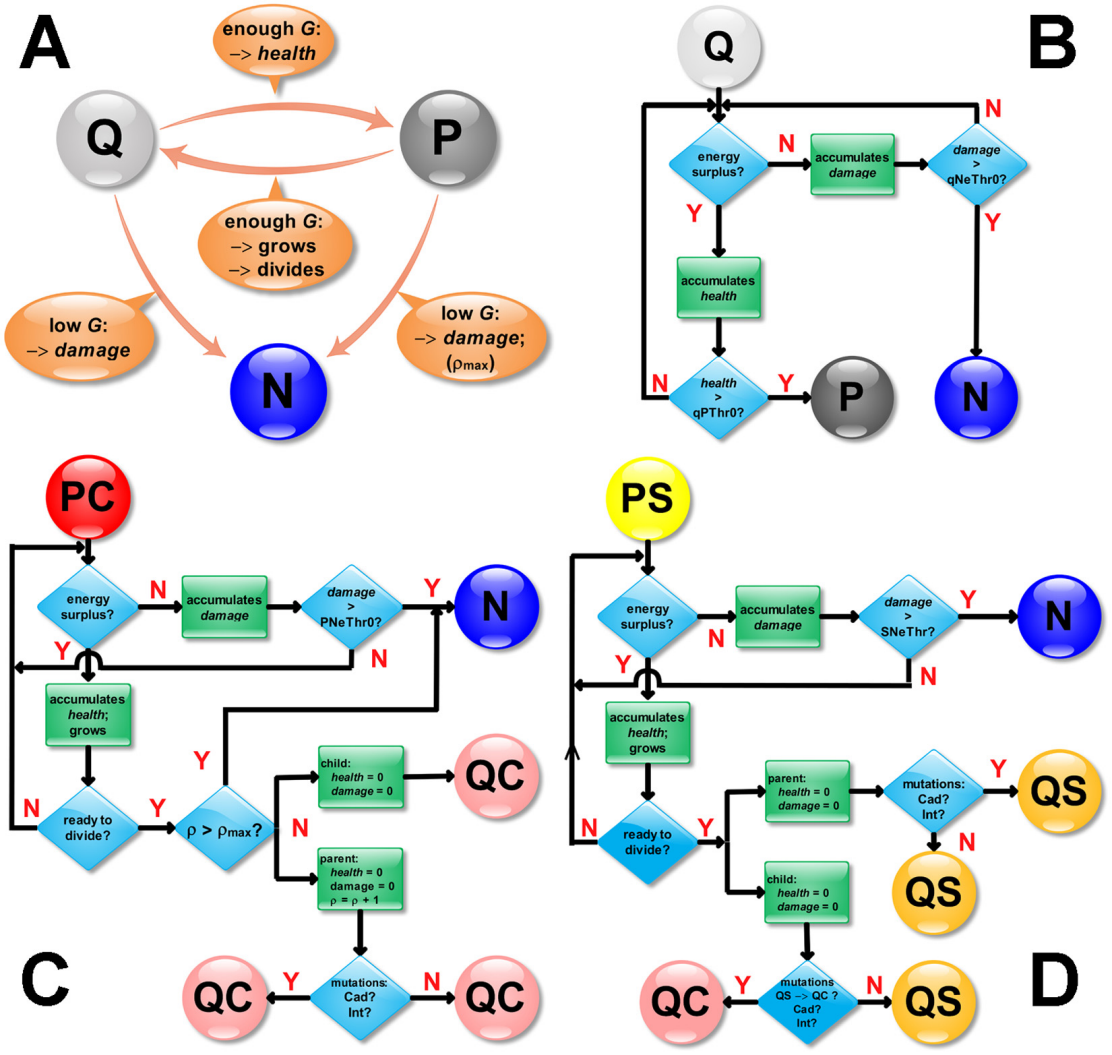
**A**) Model cell-state transitions as a function of the glucose supply. As implemented in the simulation: (**B**) Flowchart for (Q)uiescent (S or C) cell transitions; (**C**) Flowchart for (P)roliferative (C)ancer cell transitions; (**D**) Flowchart for (P)roliferative (S)tem cell transitions.

Real quiescent tumor cells become proliferative only when they experience sufficient levels of nutrients for long enough periods (*refractory behavior*) [3]. We model this refractory behavior of individual quiescent-state tumor cells by requiring them to accumulate *health*, which biologically corresponds to repair of damage due to hypoxia or other harsh microenvironmental conditions. Cells that experience higher nutrient concentrations accumulate health at a faster rate, which saturates for high nutrient concentrations. Fig 1 shows how glucose supply induces transitions between model cell states and presents related flowcharts for the simulation code.

To simulate transitions between normal and damaged cell states, we calculate the accumulated starvation factor, the cumulative effect of a suboptimal level of nutrients on the cell. When the starvation factor reaches a critical threshold, tumor cells (PC, QC, PS, QS) become necrotic cells. Different cell types have different starvation thresholds, *x*_*thresh*_. To calculate the cumulative starvation factor, we periodically check the glucose concentration at the center of mass of each cell. If the concentration is lower than the cell’s *concentration threshold*, the cell accumulates starvation damage at a rate increasing with the difference between the concentration threshold and the glucose concentration, *x* = *x*_*thresh*_ −*G* according to a Michaelis-Menten function with a saturation limit, *m*:

$$\begin{array}{c} M(x)=m\frac{x}{x+k}, \end{array}$$ (18)

where *m* is the maximum uptake rate of glucose in a given cell and *k* is a Michaelis constant. Note that $M\left(k\right)=\frac{m}{2}$, $M\left(x\right)\approx \frac{m}{k}x$ for *x* ≪ *k* and *M*(*x*) ≈ *m* for *x* ≫ *k*. Table 5 shows the Python code for this function.

When, during a single MCS, the available glucose concentration is lower than *x*_*thresh*_ the cell’s actual uptake of glucose is less than its uptake at a concentration of *x*_*thresh*_. Because cell starvation occurs when the glucose concentration falls below this threshold value, we define the starvation-factor increment as:

$$\begin{array}{c} \Delta S(x)=\{\begin{array}{cc} |M(x)-M(x_{thresh})|, & \text{for}\;\;x<x_{thresh},\\ 0, & \text{for}\;\;x\geq x_{thresh}. \end{array} \end{array}$$ (19)

In an analogous way, we define a cumulative “health” factor for cells:

$$\begin{array}{c} \Delta Q(x)=\{\begin{array}{cc} M(x)-M(x_{thresh}), & \text{for}\;\;x<x_{thresh},\\ 0, & \text{for}\;\;x\geq x_{thresh}. \end{array} \end{array}$$ (20)

In our model, at each MCS we check the concentration of glucose, *G*(*x*_*COM*_), at each cell’s center of mass, then increase either the cell’s cumulative starvation factor by Δ*S* or the cell’s cumulative health factor by Δ*Q*, depending on whether the cell’s *x*_*thresh*_ is greater or less than *x*.

We then iterate over each generalized cell in the simulation and determine whether the starvation coefficient is above the type-transition threshold for that generalized-cell’s type, in which case we change the generalized-cell type to Necrotic. The parameters ip.PNeThr0, ip.QNeThr0, ip.SNeThr0 and ip.QSNeThr0 store the starvation thresholds for PCancer, QCancer, PStem and QStem generalized-cell types respectively. For quiescent cells (generalized-cell types QCancer and QStem) we also check the health factor. If it is above the threshold for that generalized-cell state (ip.QPThr0 and ip.QSSThr0, respectively), we change the generalized-cell state to PCancer and PStem respectively. After a cell-type transition, we reset the health factor to zero. Table 6 presents the Python implementation of the model’s cell-type transitions, based on the starvation coefficients calculated in Table 7 in the Appendix.

Parameter Estimation: We assume that cells starve and accumulate damage when the Glucose concentration drops below 0.5 mM (0.032 fmol/voxel) (compare to [25, 26]). We calculate PNeThr0 and SNeThr0 based on the assumption that the cells die if they experience a glucose concentration of zero for 24 hours (damage accumulates at its highest rate for 24 hours = 102 MCS) [25, 26]. We assume that QCancer and QStem cells are more resistant to starvation than proliferating cells, so that both QNeThr0 and QSNeThr0 are twice as large as PNeThr0. We calculate QPThr0 and QSSThr0 based on the assumption that QCancer and Qstem cells that experience 5mM glucose for 24 hours switch to a proliferative state.

**Cell Growth and Cell Death**

Biomodel: A subpopulation of tumor cells proliferates when it has access to sufficient levels of nutrients. In our model, only PC and PS generalized cells grow and divide (via mitosis) once they reach their doubling volume. PC and PS generalized cells grow by consuming glucose. PC and PS generalized cells grow only when the glucose supply is above a threshold and these generalized cells grow faster for higher glucose levels. Necrotic generalized cells shrink at a constant rate, which is independent of the level of nutrients. In our model, once a generalized cell becomes Necrotic it cannot change back into any other generalized-cell type. Quiescent cancer cells (QC) and quiescent stem-like cells (QS) neither grow nor shrink. A generalized-cell’s volume and surface constraint parameters remain unchanged from the time its state changes to QC or QS.

We implement generalized-cell growth and shrinkage by manipulating generalized-cells’ *V*_*i*_s, *S*_*i*_s and *λ*s in Eq (1).

Proliferating cancer cells (PC) and proliferating stem (PS) cells grow at a rate:

$$\begin{array}{c} \Delta V^{i}=k\theta (G({\vec{x}}_{COM}^{i})-G_{thresh})[G({\vec{x}}_{COM}^{i})-G_{thresh}], \end{array}$$ (21)

where Δ*V* ^*i*^ is the increase in the target volume of generalized cell *i* per MCS. ${\vec{x}}_{COM}^{i}$ is the center of mass of generalized cell *i* and $G\left({\vec{x}}_{COM}^{i}\right)$ is the glucose concentration at this point. *G*_*thresh*_ is the minimum glucose concentration which allows cells to grow. *k* is the growth speed and *θ* the Heaviside step function:

$$\begin{array}{c} \theta (x)=\{\begin{array}{cc} 0, & \text{for}\;\;x\leq 0\\ 1, & \text{for}\;\;x>0 \end{array}. \end{array}$$ (22)

We update the generalized-cells’ target volumes once per MCS. To maintain the generalized-cells’ surface-to-volume ratios, we adjust the generalized-cells’ target surface area as:

$$\begin{array}{c} S^{i}=q_{s,v}\sqrt{V^{i}}, \end{array}$$ (23)

where *q*_*s*, *v*_ = 4 is a scaling factor derived on the assumption that cells in the simulation should be nearly circular in shape. In our 2D simulations, the relationship between surface area and volume for a circular generalized cell is:

$$\begin{array}{c} S=\sqrt{4\pi }\sqrt{V}. \end{array}$$ (24)

Necrotic generalized cells shrink and disappear by reducing their target volumes at a constant shrinkage rate until their target volumes reach 0:

$$\begin{array}{c} \Delta V_{Necrotic}^{i}=-\min(\delta_{V}^{Necrotic},V^{i}), \end{array}$$ (25)

where $\delta_{Necrotic}^{V}=0.05$ is the constant shrinkage rate for necrotic cells and *V* ^*i*^ is the current target volume of the Necrotic generalized cell. Once a generalized-cell’s target volume reaches 0, it will disappear as neighboring generalized cells overwrite its voxels. Table 8 shows the Python implementation for these mechanisms.

Parameter Estimation: We assume that PC and PS generalized cells that experience 5mM glucose take 24 hours to reach their doubling volume and divide. We set *g* in Eq (21) to 0.34 fmol/voxel and set *k*(incvol) = 0.2 so it increases the generalized-cells’ target volume by 16 voxels in 24 hours. To allow elongated generalized-cell shapes and prevent alignment of generalized-cell boundaries along the cell-lattice’s symmetry axes, we set *q*_*s*, *v*_ = 4, slightly higher than $\sqrt{4\pi }$ in Eq (24).

**Mitosis, Senescence and Mutation**

Biomodel: When a growing cell reaches its doubling volume, it undergoes mitosis. Depending on the type of cell undergoing mitosis and the number of cell cycles it has completed, the two daughter cells may change their types and cadherin and integrin expression levels.

Mitosis splits cells into two daughter cells of roughly equal volumes. The Python code in Table 9 sweeps through all generalized cells once per MCS to identify generalized cells that should divide.

In our model, we refer to a cell just before mitosis as a *parent cell*. Mitosis divides the voxels of the parent generalized cell roughly equally between a generalized cell which keeps the identity of the parent generalized cell and a new *daughter generalized cell*. While this usage differs from the biological terminology, it reflects the actual bookkeeping in the simulation. Mitosis resets the parent and daughter generalized-cells’ target volumes and target surface areas to their reference values, and their cumulative damage and health to 0. In the model only two generalized-cell types grow and divide: PC, PS. A senescence counter, *n*_*d*_, counts the number of divisions a PC or QC generalized cell has undergone since its transition from a PS generalized cell. We assume that cancer cells typically undergo a maximum number of divisions *m*_*d*_. On each division of a PC parent generalized cell we pick *R* from a Gaussian distribution *r*:

$$\begin{array}{c} r(x;\;m_{d},\delta_{d})=\frac{1}{\delta_{d}}\phi (\frac{x-m_{d}}{\delta_{d}}), \end{array}$$ (26)

where *r* (*x*; *m*_*d*_, *δ*_*d*_) is the Gaussian probability distribution of variable *x* with mean *m*_*d*_ and standard deviation *δ*_*d*_ and

$$\begin{array}{c} \phi (x)=\frac{1}{\sqrt{2\pi }}e^{-\frac{x^{2}}{2}}. \end{array}$$ (27)

If *n*_*d*_ > *R* both the parent and daughter generalized cells become Necrotic. For PC generalized cells, *m*_*d*_ = 8 and *δ*_*d*_ = 2.

When a PS generalized cell divides, the parent generalized cell becomes a QS generalized cell and the daughter generalized cell has a probability of 1 – ip.probstem of becoming a QC generalized cell and of ip.probstem of becoming a QS generalized cell. We normally set ip.probstem = 0.2. As the “Cell-State Transitions” section describes, high nutrient availability induces quiescent stem cells (QS) to become proliferating stem generalized cells (PS). Our simulation implements the senescence and stem generalized cell → cancer generalized cell transition using the Python updateAttributes function in Table 10.

CompuCell3D calls the updateAttributes function immediately after a parent generalized cell divides. The function first resets the parent and daughter generalized-cells’ *V*_*i*_s, *S*_*i*_s and *λ*s. ip.V0 stores their reference target volume and ip.LBD_V0 and ip.LBD_S0 store their reference *λ* ^*vol*^ and *λ* ^*sur*^ (see Eq (1)).

During mitosis, we also simulate random changes in the surface expression of adhesion molecules. Each parent generalized cell has a small probability (*p*_*m*_ = 0.1 stored in ip.probmut) of randomly changing its adhesion-molecule expression levels. We scale expression levels of cadherins and integrins to the range 0 to 16. After mitosis, we draw a number *R* from a gaussian distribution *r* (*x*;*j*_*am*_, *δ*_*am*_), where *j*_*am*_ is the current adhesion-molecule expression level for the parent generalized cell and *δ*_*am*_ is the standard deviation of the gaussian distribution. In our simulations, we set *δ*_*am*_ = 2.0. We store the cadherin expression level in the jcadh variable and the integrin expression level in jint. ip.cadhstdev stores the value of *δ*_*am*_, which we assume is the same for cadherins and integrins.

When *R* is within the allowed expression-level interval ([0, 16]) we set:

$$\begin{array}{c} j_{am}=R, \end{array}$$ (28)

otherwise, we reject the mutation. Our model varies only the levels of cadherin and integrin and not the level of fibronectin (FN)—*i.e*., the adhesion molecule associated with Medium. Daughter generalized cells inherit their parent generalized-cell’s adhesion molecule expression levels. Table 11 shows Python code implementing this mutation mechanism.

Parameter Estimation: We limit the AdhesionMoleculeDensity of both Int and Cad to values between 0 and 16 because this range produces both positive (cohesive) and negative (invasive) surface tensions and minimizes cell-lattice artifacts that can occur for large, positive surface tensions. Our algorithm rejects adhesion-molecule expression-level changes if they would cause the adhesion-molecule expression level to fall outside the allowed interval, which eliminates bias towards the end-values of the interval. Setting expression levels that fall outside the allowed interval to end-values causes a bias towards the end-values of the interval. For any initial value, in the absence of selection, random mutation leads the population average to drift towards the middle of the interval. Distinguishing such random-walk drift from evolution due to selection pressure is difficult, so we set the initial adhesion-molecule expression levels to 8, the middle of the allowed interval.

To facilitate simulation of the cell processes and behaviors described in this paper our simulations include modules which the center of mass and set of voxels belonging to each generalized cell. Appendix A. (Table 12) shows CC3DML implementation of these modules.

As an initial condition, we place an 8^2^-voxel quiescent stem (QS) generalized cell in the middle of the cell lattice. Table 13 shows the CC3DML implementation of this initial condition.

Table 14 shows the CC3DML to define the cell-lattice dimensions (<Dimensions>), type of boundary conditions (<Boundary_x>, <Boundary_y>), average amplitude of cell membrane fluctuations (<Temperature>), neighbor range used to pick source and target voxels for voxel-copy attempts (<NeighborOrder>), simulation duration in MCS (<Steps>) and the seed for random number generator (<RandomSeed>).

## Results

### Size and Shape Dynamics

**Fig 2:**
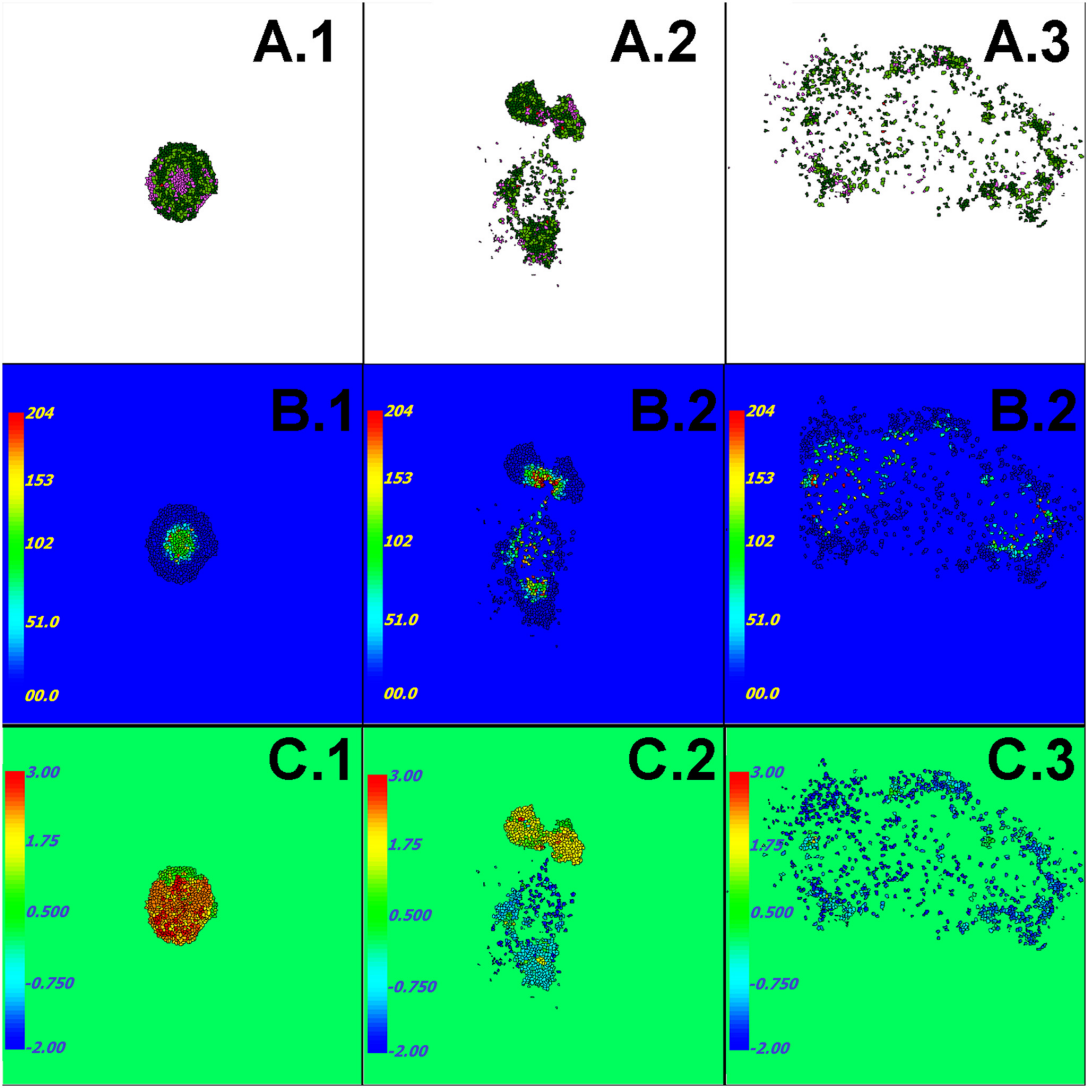
Simulation snapshots. Row A) Generalized-cell type: dark green: QCancer, light green: PCancer, dark red: QStem, light red: Pstem, Light purple: Necrotic. Row B) Accumulated damage due to starvation. Row C) Surface tension between tumor generalized cells and Medium, see Eq (12). Times: first column: ∼ 40 days, second column: ∼ 1 year, third column: ∼ 3 years. CompuCell3D code that we used to generate this figure can be downloaded from www.compucell3d.org/Models. See also Table 1 for the list of parameters used to generate this figure.

**Fig 3:**
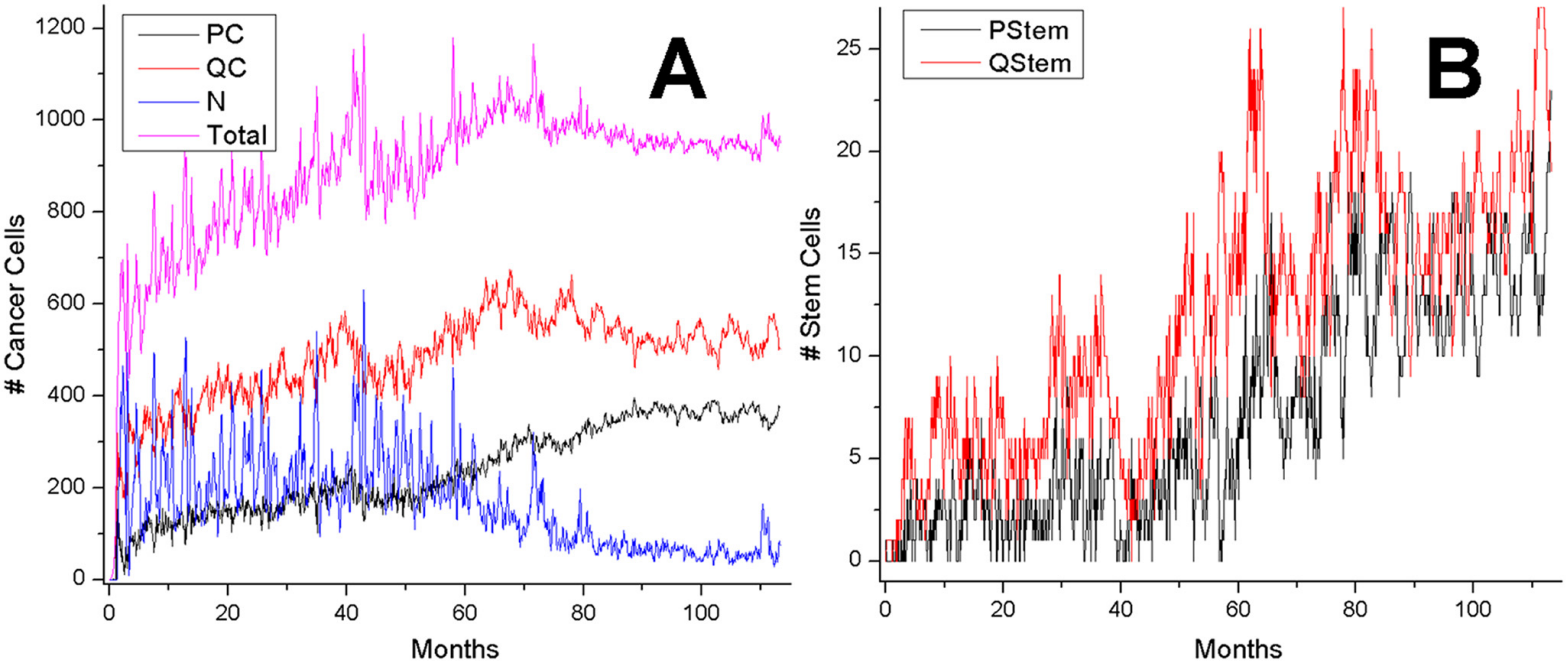
Tumor generalized-cell populations by generalized-cell type as a function of time (each plot shows results from a single simulation replica). The fraction of stem-like cancer generalized cells increases steadily in all simulations.

In our simulations, the initial cancer stem (QS) generalized cell grows into a cohesive solid tumor that reaches a maximum diameter of about 500 *μ*m during the first simulated month. During the next simulated 10 days (day 30 to day 40), tumor generalized cells at the center of the tumor die and form a necrotic core that is about 250 *μ*m in diameter (Fig 2A.1 and 2B.1). At this stage, some PS generalized cells at the periphery also die through senescence after reaching their maximum number of divisions. Since we do not model angiogenesis, the total amount of Glucose the Medium supplies limits the diameter of a compact tumor to under 500 *μ*m. This nutrient-limited regime lasts between four and twelve simulated months. As tumor generalized cells progressively become less cohesive (due to mutations accumulated during mitosis) (Fig 2A.2), the tumor becomes less spherical and elongates, usually leading to splitting of the tumor into two clusters that are nearly equal in size. The less cohesive cluster then usually outgrows the more cohesive cluster, because its elongated shape has more contact area with Medium, which allows higher rates of Glucose transport into the cluster (Fig 2B.2). Any clusters that do not contain stem generalized cells eventually disappear. Tumor generalized cells become potentially invasive if they express low levels of cadherin and high levels of integrin. When such invasive generalized cells come to the edge of their cluster and contact the Medium, they can leave their clusters and invade the Medium (Fig 2A.2). Generalized cells that have invaded the Medium proliferate faster than those remaining in a cluster (see population plots in Fig 3A–3B), since glucose levels outside clusters are significantly higher than levels inside or at the periphery of clusters. Thus the spreading invasive cells eventually outcompete nearby tumor because they have higher probability of survival than generalized cells in a larger cluster.

**Table 1:** Reference parameter set. The entire model is available at www.compucell3d.org/Models—listed as *Emergent Stratification Model*. For an explanation of the effective units of these parameters see text.

**Parameter description**	**Value**
Lattice Dimension	500x500x1
Cell Mambrane Fluctuation (*T*_*m*_)	50
Voxel Copy Neighbor Order	3
Simulation Duration (in MCS)	1000001
Initial density of FN molecule in Medium, see Eq (8)	16.0
Initial density of Cad molecule in PCancer, see Eq (8)	8.0
Initial density of Int molecule in PCancer, see Eq (8)	8.0
Initial density of Cad molecule in QCancer, see Eq (8)	8.0
Initial density of Int molecule in QCancer, see Eq (8)	8.0
Initial density of Cad molecule in PStem, see Eq (8)	8.0
Initial density of Int molecule in PStem, see Eq (8)	8.0
Initial density of Cad molecule in QStem, see Eq (8)	8.0
Initial density of Int molecule in QStem, see Eq (8)	8.0
Cad-Cad binding parameter—*k*_*m*, *n*_, see Eq (8)	2.0
Int-FN binding parameter—*k*_*m*, *n*_, see Eq (8)	0.2
Glucose diffusion constant, see Eq (15)	13500.0
Glucose decay constant, see Eq (15)	0.45
QStem glucose max. uptake rate, see Eq (14)	1.69
QStem Michaelis-Menten-Coefficient, see Eq (14)	0.00256
PStem glucose max. uptake rate, see Eq (14)	2.25
PStem Michaelis-Menten coefficient, see Eq (14)	0.00256
QCancer glucose max. uptake rate, see Eq (14)	1.69
QCancer Michaelis-Menten-Coefficient, see Eq (14)	0.00256
PCancer glucose max. uptake rate, see Eq (14)	2.25
PCancer Michaelis-Menten coefficient, see Eq (14)	0.00256
Uniform glucose secretion rate—Medium	0.145
Initial target volume (ip.Vo)	16.0
Initial target surface (ip.S0)	16.0
Lambda volume—$\lambda_{\sigma }^{vol}$ (ip.LBD_V0)	15.0
Lambda surface—$\lambda_{\sigma }^{sur}$ (ip.LBD_S0)	5.0
Growth-rate factor (ip.incvol)—*k* see Eq (21)	0.2
Shrinkage-rate factor (ip.decvol)—$\delta_{V}^{Necrotic}$ see Eq (25)	0.01
Cancer-cell doubling volume (self.volmaxmit)	32
Stem-cell doubling volume (self.Svolmaxmit)	32
Maximum number of divisions (ip.maxdiv)	8
Probablilty of daughter cell becoming stem cell (ip.probstem)	0.2
Probability of cadherin mutation—*p*_*m*_ (ip.probmut)	0.1
Width (*δ*_*d*_ = *δ*_*am*_) of cadherin value distribution function, see Eq (26)	2.0
Initial relaxation threshold (ip.MCSThr)	50
Concentration threshold for PCancer cells’ growth (ip.PGrThr0)	0.032
Concentration threshold for PStem cells’ growth (ip.SGrThr0)	0.032
PCancer-to-Necrotic-transition damage threshold (ip.PNeThr0)	102.0
QCancer-to-Necrotic-transition damage threshold (ip.QNeThr0)	204.0
PStem-to-Necrotic-transition damage threshold (ip.SNeThr0)	408.0
QStem-to-Necrotic-transition damage threshold (ip.QSNeThr0)	916.0
QCancer-to-PCancer-transition health threshold (ip.QPThr0)	79.5
QStem-to-PStem-transition health threshold (ip.QSSThr0)	79.5
Damage accumulation threshold (ip.GluD)	0.0032

For the reference set of parameters, predominantly dispersed, invasive generalized cells become dominant after about 3 simulated years (Fig 2A.3)—see section “Parameter Effects on Tumor Growth and Evolution” for detailed studies of the parameter space. After this stage, small clusters (less than 20 cells) continuously form and disintegrate, as stem-like generalized cells leave their clusters or die due to rapid accumulation of damage. Even though we did not vary the fraction of stem-like offspring of stem-like generalized cells or allow this fraction to evolve in this paper, the fraction of stem-like generalized cells in the total population gradually increases with time. We will present our studies of the effects of evolutionary change on the fraction of stem-like generalized cells in a future paper.

### Starvation and Evolution of Cell Adhesion

**Fig 4:**
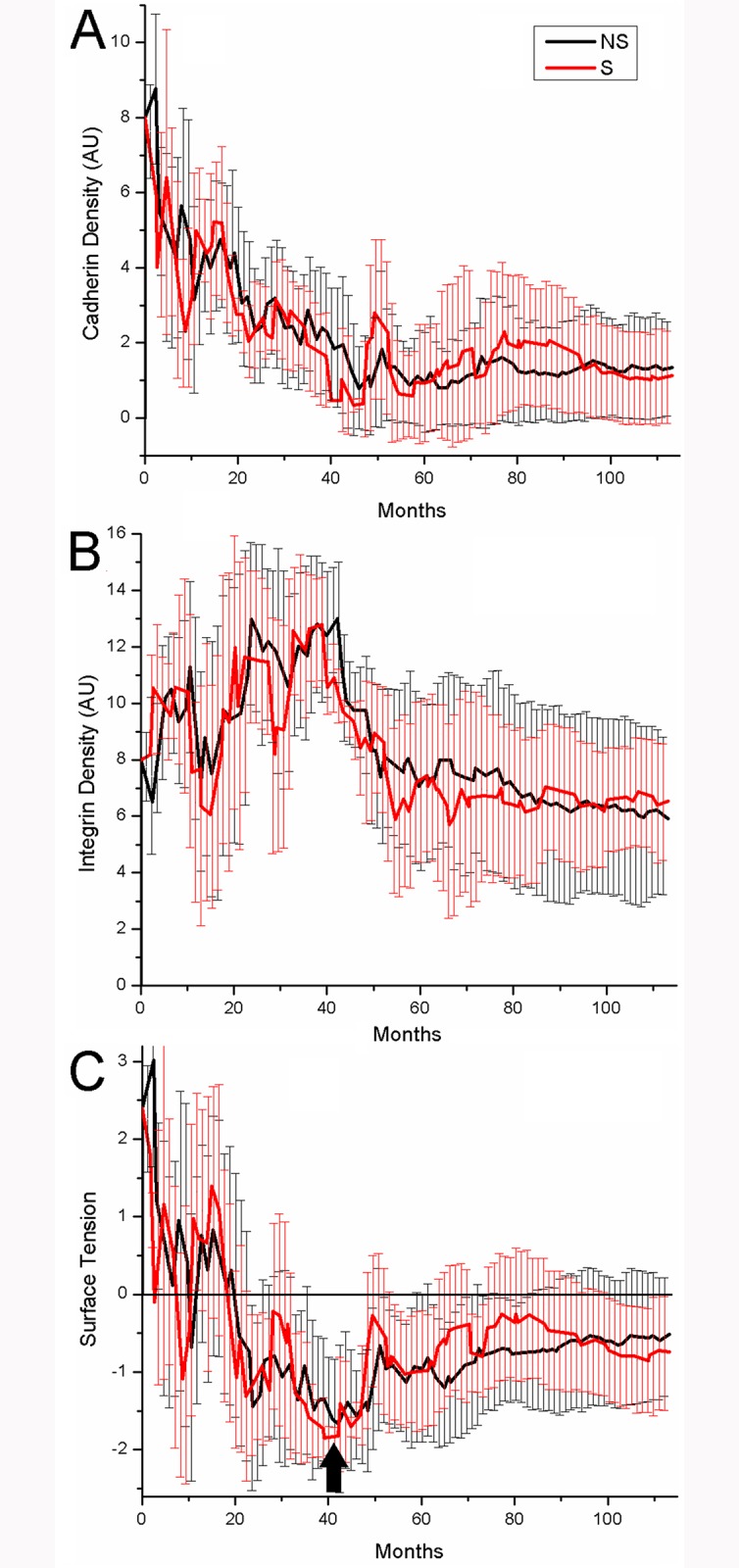
Evolution of typical generalized-cell cohesivity vs. time. A) Average density of cadherin (Cad) of tumor generalized cells in arbitrary units (AU) vs. time. B) Average density of integrin (Int) of tumor generalized cells in arbitrary units (AU) vs. time. C) Average surface tension between tumor generalized cells and Medium vs. time.

In our model, invasion of Medium is a winning strategy for generalized cells. Generalized cells gain invasive behaviors in two key phases. In the initial phase, as long as generalized cells remain in clusters, differential cell adhesion leads to cell-sorting in which generalized cells expressing lower levels of cadherins and higher levels of integrins (relative to the typical levels in the cluster) move to the surface of the cluster and generalized cells expressing higher levels of cadherins (relative to typical values in the cluster) move towards the center of the cluster (the black arrow in Fig 4C). As generalized cells move towards the center of the cluster, they cease to proliferate, starve and eventually die. Thus generalized cells which remain on the surface of the cluster proliferate more rapidly than those in the interior of the cluster, selecting for the least cohesive generalized cells in the cluster. Since a generalized cell’s cadherin level has a greater effect on its radial position in the cluster than its integrin level, initial selection predominantly favors generalized cells with decreased cadherin levels (Fig 4A). Because starvation selects for lower relative adhesion between generalized cells, the typical cadherin level of the generalized cells in the cluster decreases continuously until, in the second phase, the least cohesive generalized cells (those with high levels of integrin and low levels of cadherin) in the cluster are able to migrate out of the cluster. This invasive phenotype corresponds to a negative surface tension between Medium and tumor generalized cells (Fig 2C.2, Fig 4B–4C). As we discussed in section “Size and Shape Dynamics”, invasive generalized cells then come to dominate the tumor-cell population, and spread throughout the Medium.

## Parameter Effects on Tumor Growth and Evolution

Our model of emergent tumor stratification has about 50 parameters. Some parameters values are available in the cancer literature, others are not, including the probability of a random mutation of a generalized-cell’s cadherin expression and the distribution of changes in cadherin expression on mutation. In any case, because we are simulating a much smaller number of generalized cells than the number of cells we would find in a real tumor, we must greatly increase the probability of mutation per generation and the magnitude of the typical phenotypic consequence of a mutation, if we wish to observe a significant evolution of the phenotype. Since the goal of this work is to examine how specific types of selection pressure contribute to the emergence of specific tumor morphologies, we care more about relative rates of mutation than absolute rates and the sequence of evolutionary events rather than their absolute times of occurrence. Despite these caveats, we observe patterns of changes of cell behavior which should be robust if we were to reduce the overall rates of mutation and increase the simulation size to more realistic values. As long as all cells are sufficiently cohesive that they remain attached to the primary cluster, selection favors cells with lower relative cell-cell adhesivity and higher cell-stroma adhesivity. Because only cells with lower relative adhesivity benefit, the mean absolute adhesivity decreases until cells are able to separate from their cluster and invade the surrounding tissue. To check the robustness of this evolutionary pattern to the mutation rate and amplitude parameters, we varied the probability of cadherin-level and integrin-level mutation *P*_*m*_ and the standard deviation *δ*_*am*_ of the Gaussian probability distribution used to select the mutated cadherin and integrin expression levels (see eqs. (26) and (28)), using the same *P*_*m*_ and *δ*_*am*_ for both cadherin and integrin.

For each value of *P*_*m*_ (from the set {0.1, 0.2, 0.3}) and each value of *δ*_*am*_ (from the set {0.5, 1.0, 1.5, 2.0}) we ran 10 simulation replicas with different random seeds. We then correlated *P*_*m*_ and *δ*_*am*_ values with the typical behavior of the replicas. As we would expect, larger *P*_*m*_ and *δ*_*am*_ decreased the time until the first appearance of generalized cells able to invade the surrounding stromal tissue and increased the average number of clusters at a given time. Despite these quantitative differences, the qualitative series of changes in generalized-cell and cluster properties were independent of the mutation rate and amplitude.

### Parameter Scan Methodology

To facilitate parameter scans, we developed a parameter-scan module for CompuCell3D that automatically runs multiple replicas of a simulation over a user-defined parameter space. We analyzed our parameter-scan results using Python scripts employing the 3rd-party tools Pandas (http://pandas.pydata.org/), numpy (http://www.numpy.org) and scikit-learn (http://scikit-learn.org/).

The key to efficient management of studies which require long simulations is to store sufficient information to support flexible postprocessing. Otherwise, a change to the analysis protocol can require time-consuming rerunning of all replicas in the entire simulation set. We therefore store, every 1000 MCS, a summary file (a data *snapshot*) including each generalized-cell’s index, type, volume, surface area, cadherin and integrin levels, and center-of-mass position. Although a typical simulation replica has only a few hundred generalized cells at any time, a typical run generates between 20,000 and 50,000 tumor generalized cells as cells divide and die. In addition, generalized cells frequently change type (see Fig 1). When we approximate generalized-cell-type-dependent metrics, *e.g.*, the average distance generalized cells of a given type travel over a fixed time, we bin the data for that generalized cell based on the type it had when it first appeared in a snapshot. While a recording interval of 1000 MCS is too slow for some types of measurement, it suffices for evolutionary changes, which happen on an even slower time scale.

To aggregate the behaviors of multiple simulation replicas with identical *P*_*m*_ and *δ*_*am*_, for each simulation metric we used envelop plots showing the minimum, maximum, and median metric value as a function of time. In a substantial number of cases, the behavior of a few replicas differs significantly from the behavior of a typical replica (see cluster plots for *e.g.*, *P*_*m*_ = 0.1 and *δ*_*am*_ = 1.0). However, none of these cases refutes our basic conclusions about the evolution of invasive phenotypes.

### Generalized-Cell-Type Populations

**Fig 5:**
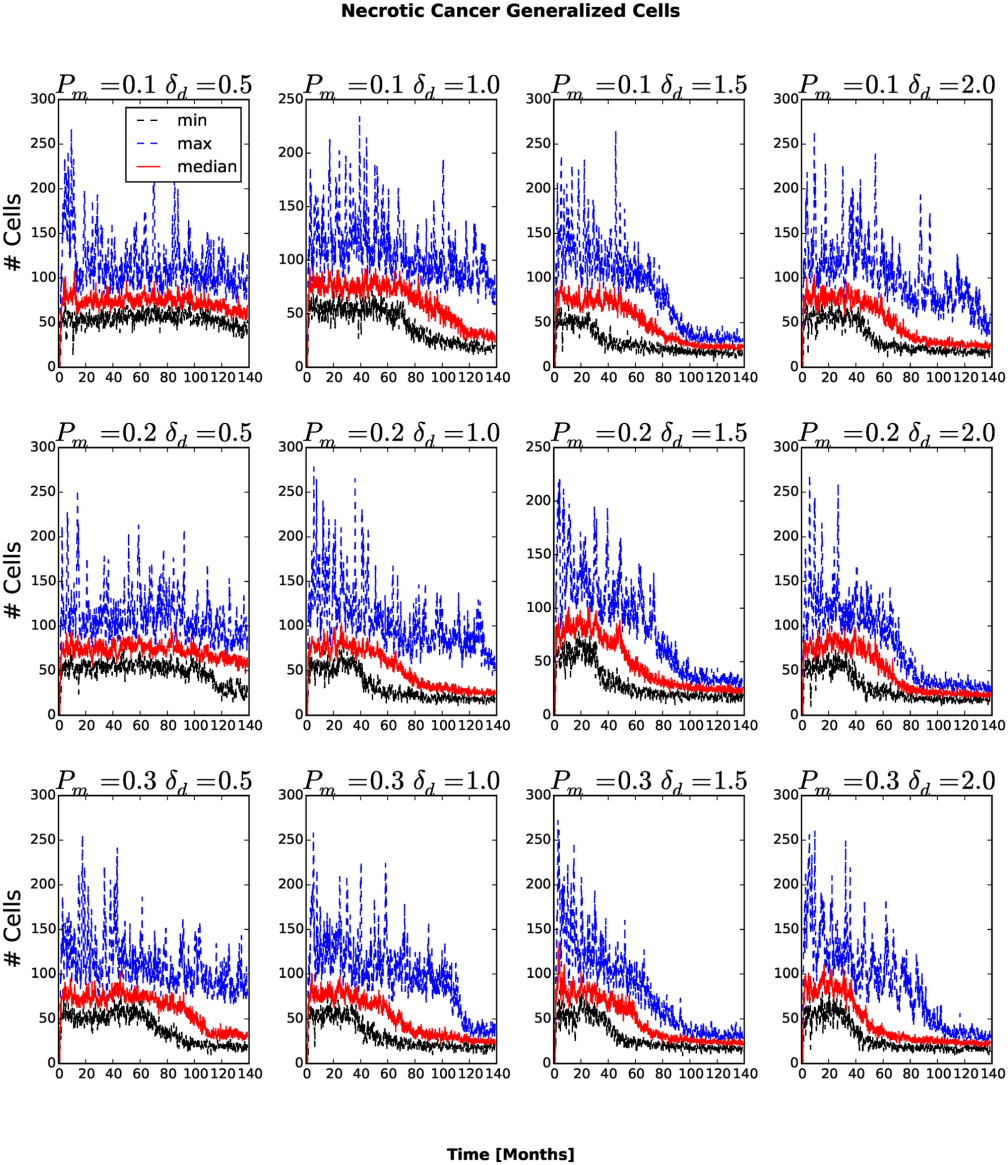
Number of Necrotic generalized cells as a function of time. In all replicas, the number of Necrotic generalized cells decreases after generalized cells begin to invade the stromal tissue.

**Fig 6:**
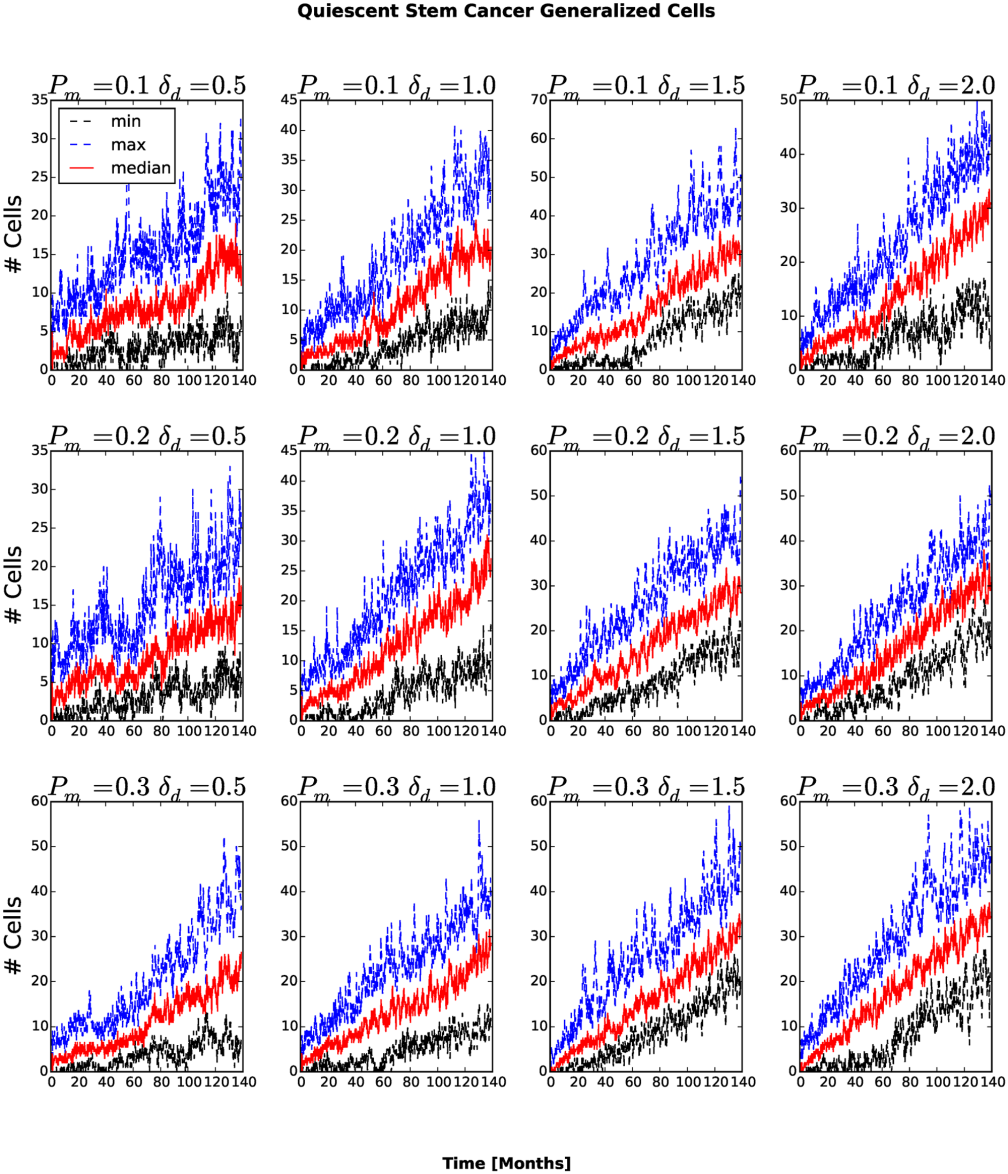
Number of quiescent cancer stem-like generalized cells as a function of time. In all replicas, the number of quiescent cancer stem-like generalized cells increases in time.

**Fig 7:**
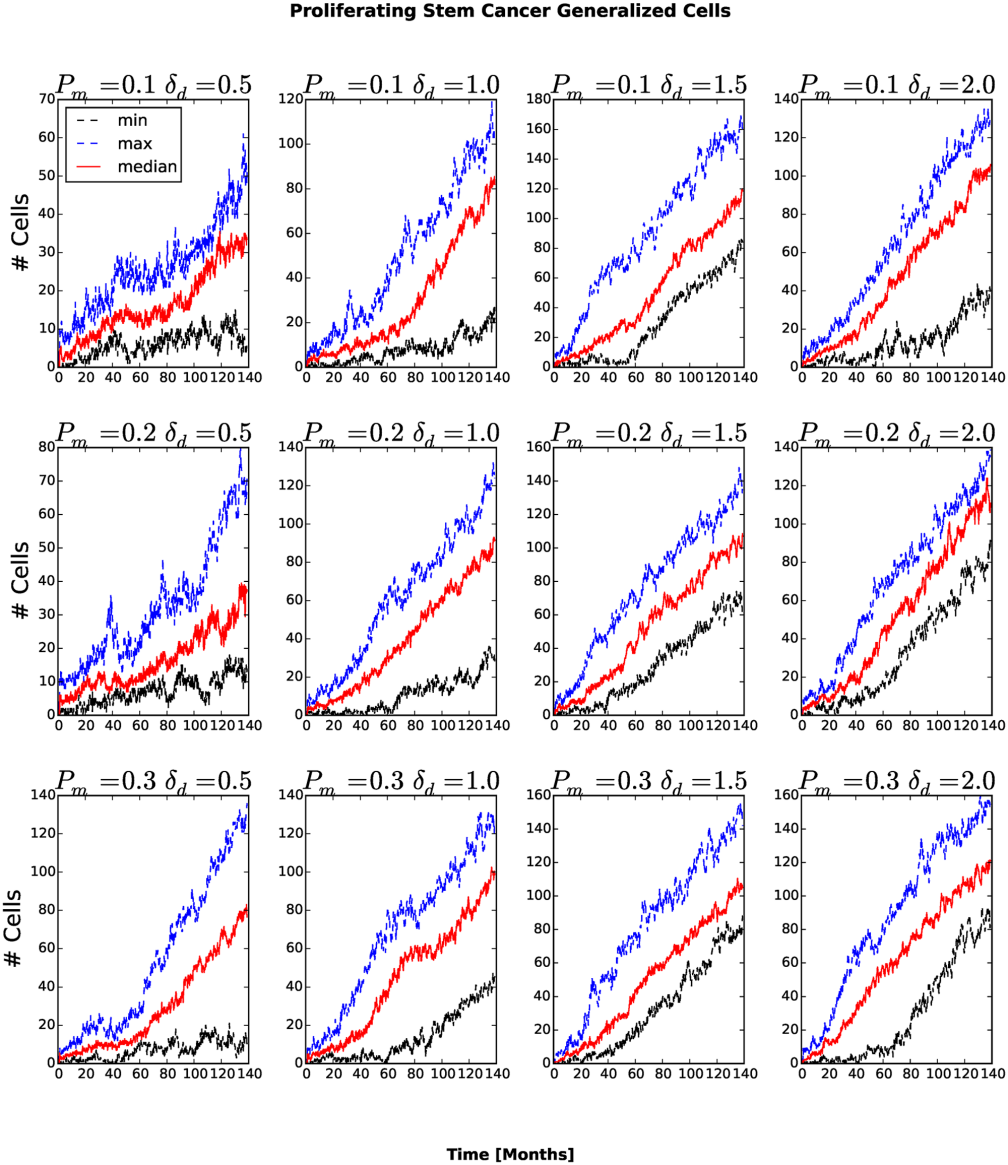
Number of proliferating cancer stem-like generalized cells as a function of time. In all cases, the number of proliferating cancer stem-like generalized cells increases in time.

**Fig 8:**
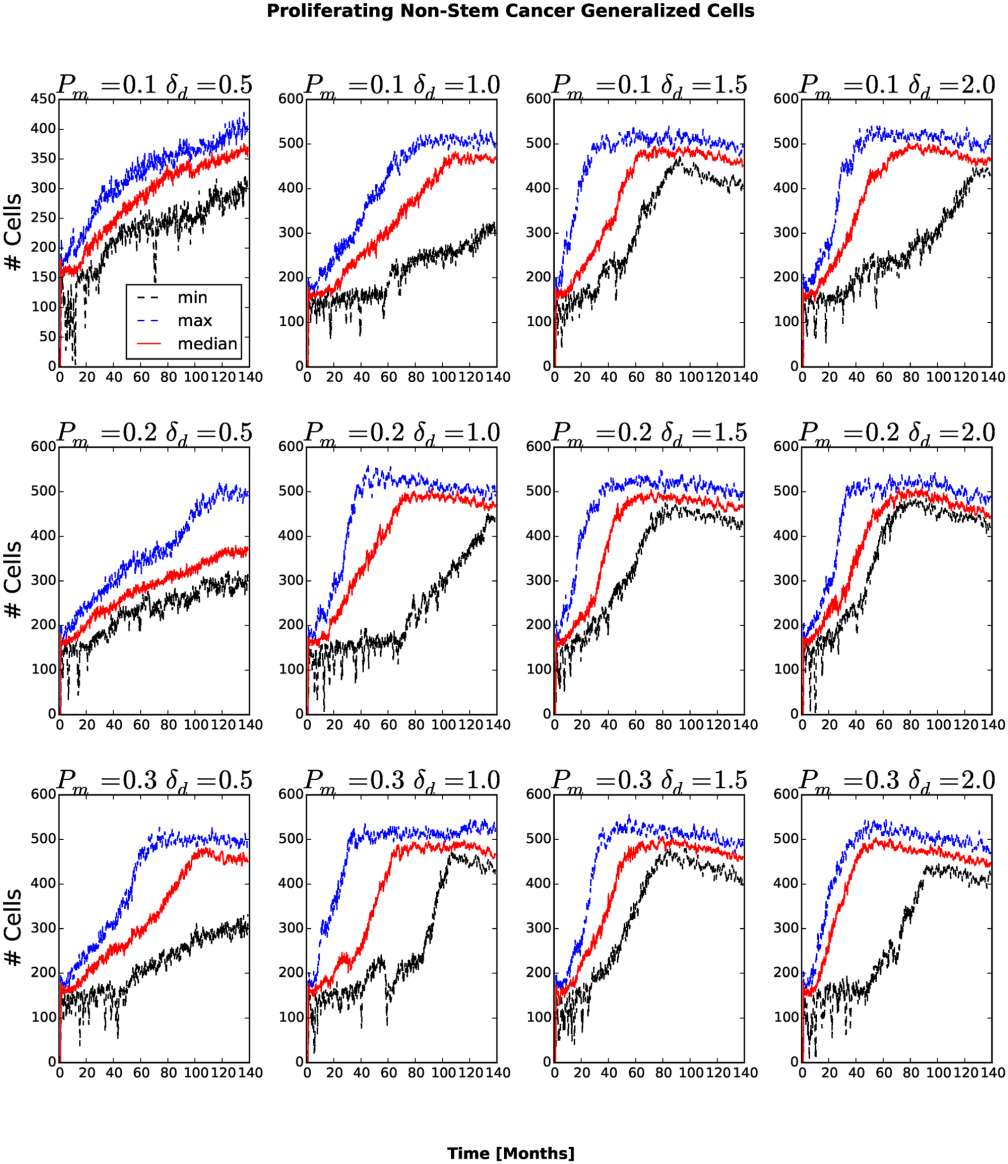
Number of proliferating non-stem cancer generalized cells as a function of time. The number of proliferating non-stem cancer generalized cells saturates and even to decrease when most generalized cells are able to invade the stromal tissue, forming numerous tumor small clusters.

**Fig 9:**
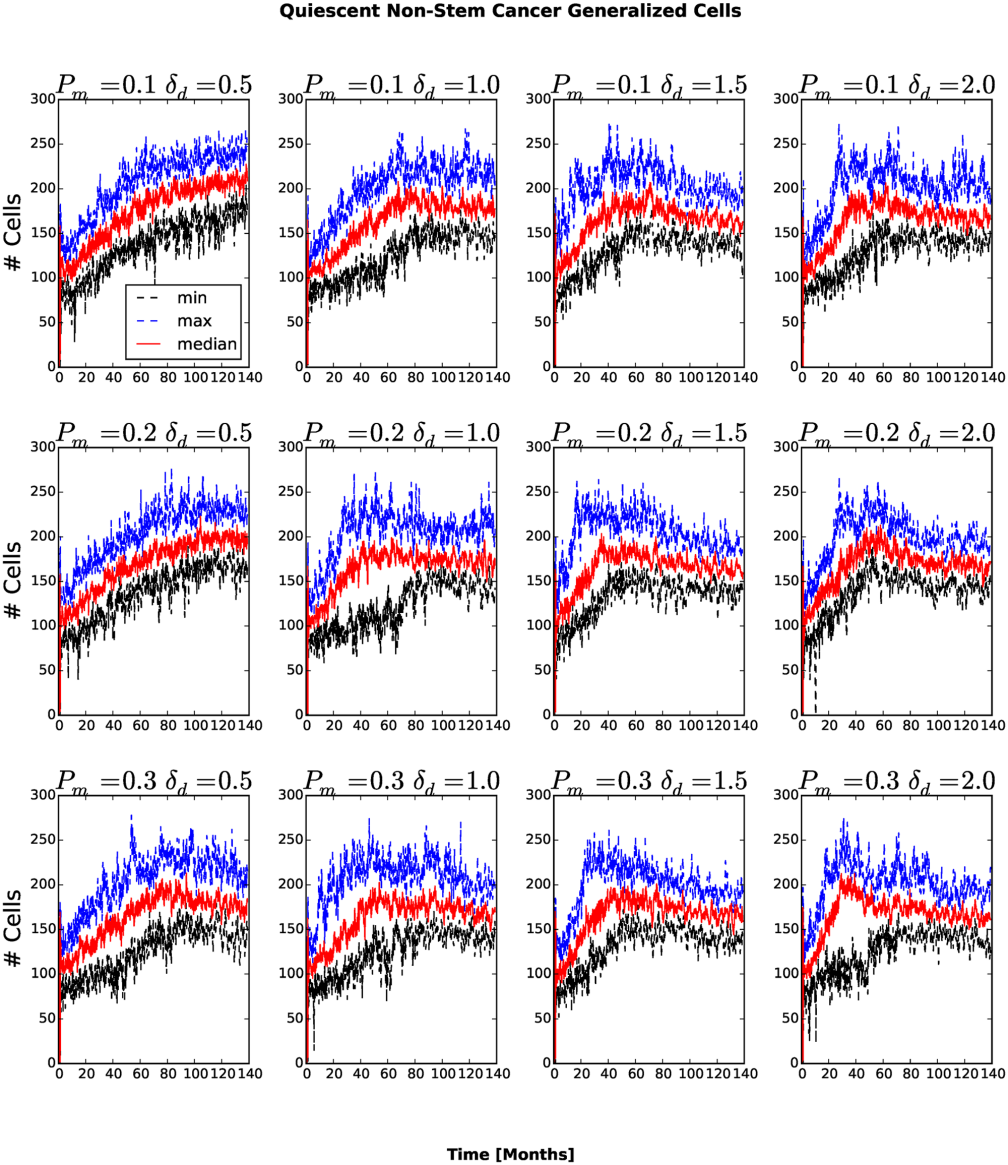
Number of quiescent non-stem cancer generalized cells as a function of time. The number of quiescent non-stem cancer generalized cells saturates and begins to decrease once a few generalized cells are able to invade the stromal tissue and the mean cluster size begins to decrease.

The number and fraction of generalized cells of each type changes with time. The number of Necrotic generalized cells increases as the cluster grows, then decreases once generalized cells begin to invade the surrounding tissue, see Fig 5. For *P*_*m*_ = 0.1, *δ*_*am*_ = 0.5 and for *P*_*m*_ = 0.1, *δ*_*am*_ = 1.0 the number of Necrotic generalized cells decreases slowly, while for other combinations of *P*_*m*_ and *δ*_*am*_ the number of Necrotic generalized cells decreases rapidly. A cluster must contain at least one stem-like generalized cell to survive, so we expect the fraction of stem-like generalized cells to increase as the number of clusters increases and the cluster size decreases. The number of stem-like generalized cells increases continuously. For *P*_*m*_ = 0.1, *δ*_*am*_ = 0.5 and *P*_*m*_ = 0.1, *δ*_*am*_ = 1.0 the number of stem-like generalized cells increases more slowly than for other combinations of parameters (see Figs. 6 and 7). As more generalized cells develop an invasive phenotype, the initial cluster gives way to many smaller clusters. Because these clusters are small, almost all generalized cells receive sufficient glucose and the number of necrotic generalized cells decreases as the number of proliferating non-stem cancer generalized cells begins to saturate (see Fig 8). We see a clear distinction if we compare the numbers of quiescent (Fig 9) and proliferating (Fig 8) non-stem cancer generalized cells. Both saturate and begin to decrease, the former as soon as a few generalized cells establish new clusters, the latter only when small clusters fill the entire tissue.

### Number of Clusters

**Fig 10:**
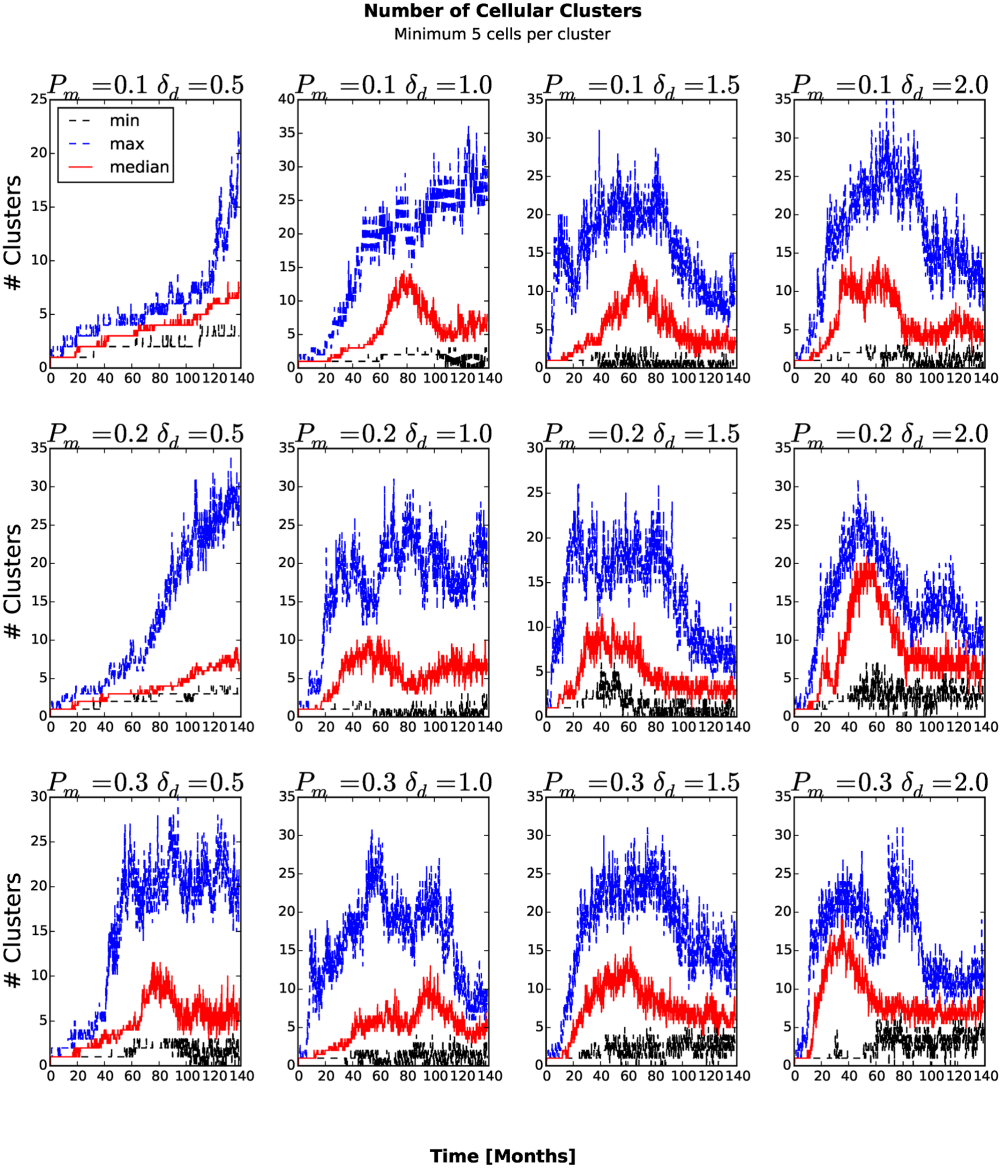
Number of clusters with at least five generalized cells as a function of time. The number of clusters increases in time once generalized cells become able to invade the stromal tissue and establish new clusters.

**Fig 11:**
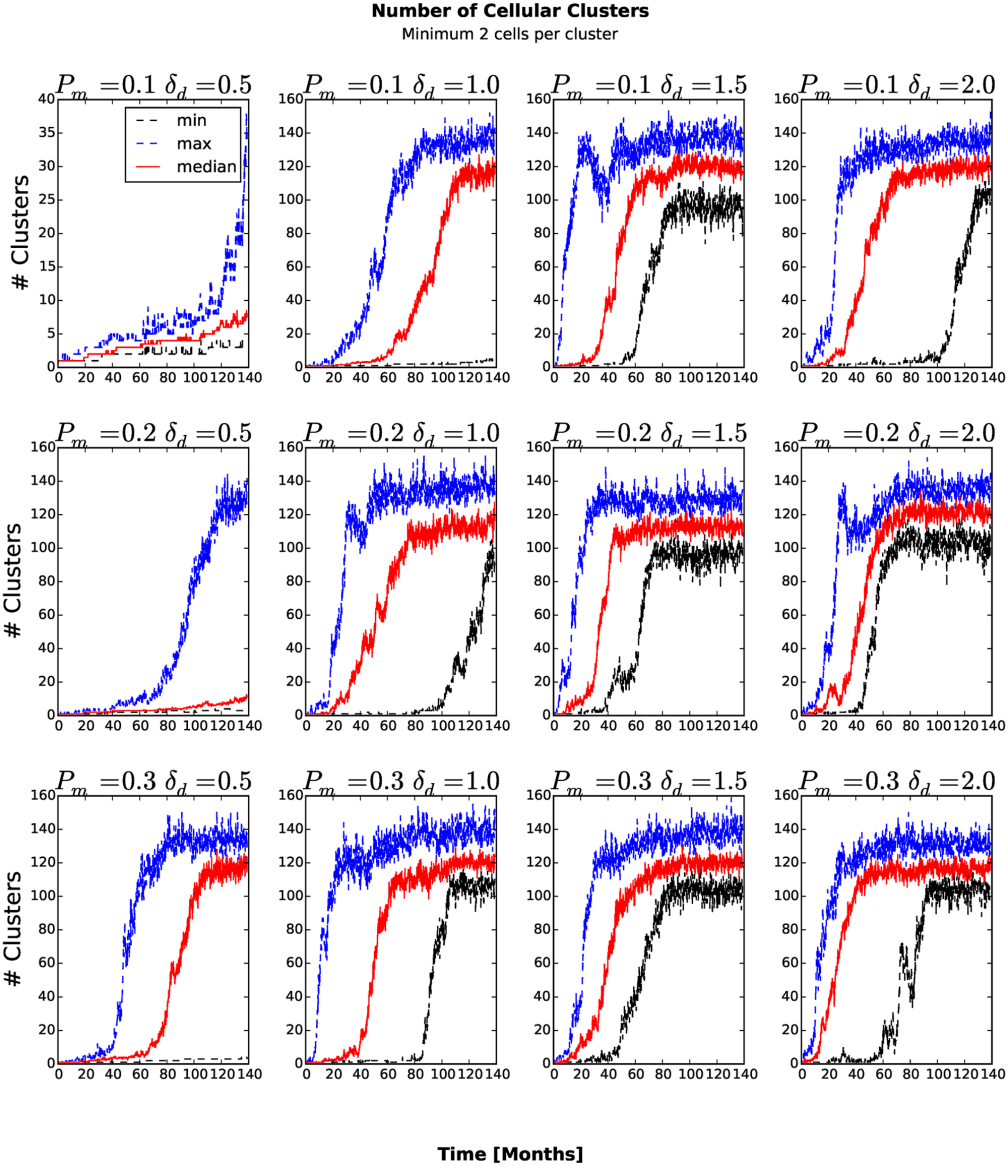
Number of transient associations of 2, 3 and 4 generalized cells and clusters of five or more generalized cells as a function of time. The number of clusters increases continuously until it reaches the maximum the available space permits.

**Fig 12:**
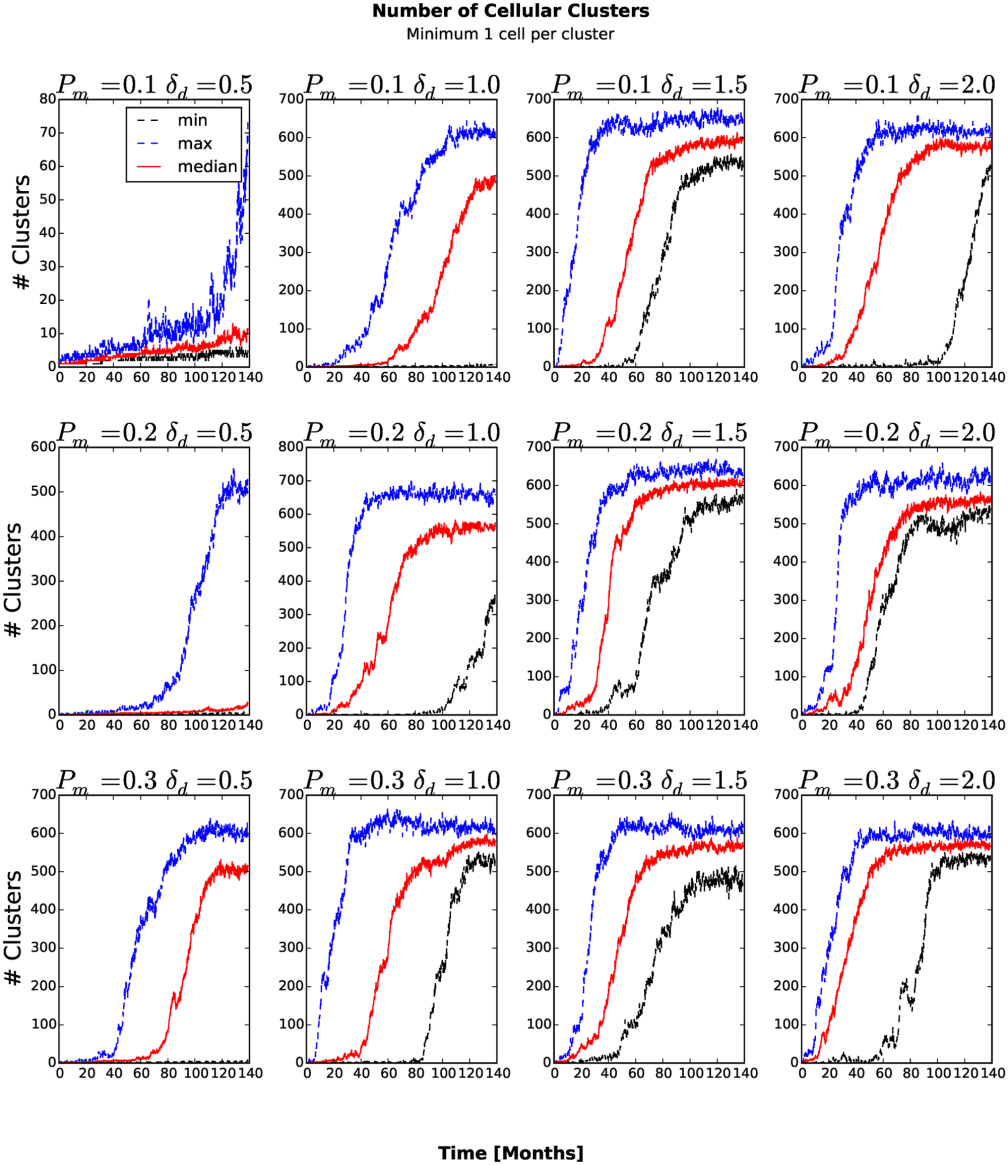
Number of isolated generalized cells, transient associations of 2, 3 and 4 generalized cells and clusters of five or more generalized cells as a function of time. The number of clusters increases continuously until it reaches the maximum the available space permits.

To count the number of generalized-cell clusters, we used the DBSCAN algorithm [27] in the scikit-learn Python package (http://scikit-learn.org). DBSCAN views clusters as areas of high density, separated by areas of low density. We defined a cluster as a group of at least five generalized cells whose centers of mass are at most 6 voxels from their nearest cluster-mate (Fig 10) and configure DBSCAN algorithm accordingly. The number of clusters increases in time once generalized cells become able to invade the stromal tissue and establish new clusters. If we define the aggressiveness of the tumor as the rate at which new clusters develop, the aggressiveness increases for larger *P*_*m*_ and *δ*_*am*_. For many combinations of *P*_*m*_ and *δ*_*am*_, the number of clusters eventually peaks and then decreases when medium-sized clusters split into isolated generalized cells and transient associations of fewer than five generalized cells. We can understand this second transition if we remember that the mean motility of the generalized cells increases continuously in time. When this motility is too small, all of the generalized cells remain condensed in a single cluster. For larger motilities, the large cluster breaks up into multiple medium-sized clusters. However, still larger motilities lead to a second phase transition in which all clusters become unstable, reducing the number of clusters (5 or more generalized cells separated a COM-to-COM distance of at most 6 voxels) that DBSCAN algorithm detects. If we also count transient associations of 2, 3 and 4 generalized cells (Fig 11) and also include isolated generalized cells as separate clusters (Fig 12), the number of clusters increases and eventually saturates, when it reaches the maximum the available space permits.

**Fig 13:**
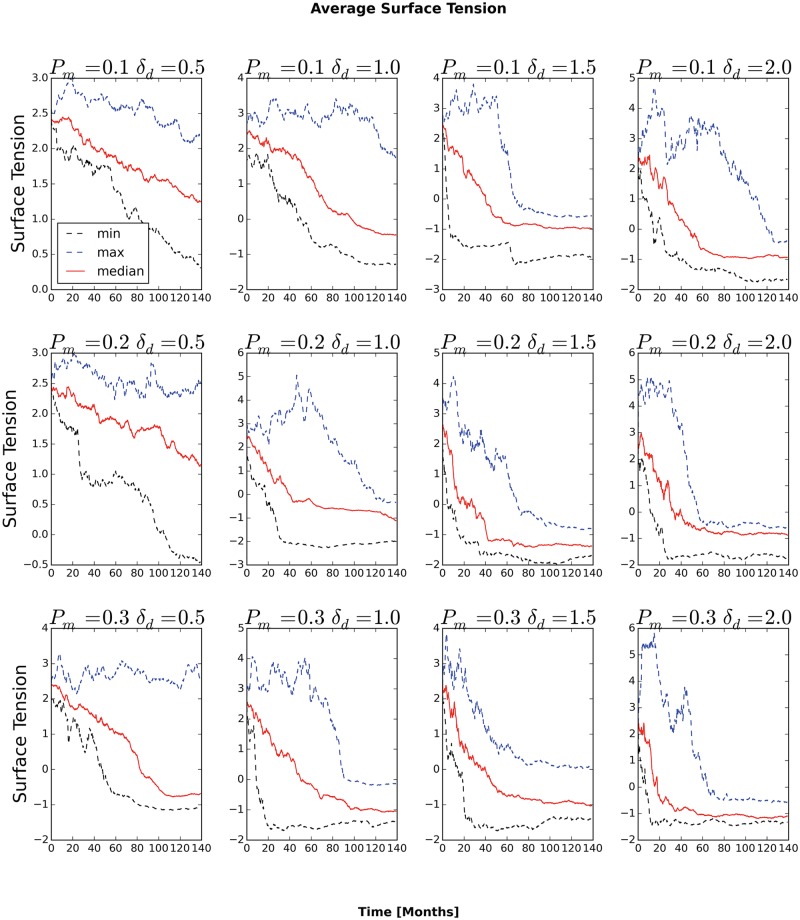
Average surface tension of tumor cells as a function of time. Only simulations with *P*_*m*_ = 0.1 and *δ*_*am*_ = 0.5 or *δ*_*am*_ *m* = 1.0 have positive average surface tension at all times. In all other cases, the average surface tension eventually drops below zero and the generalized cells invade the stromal tissue.

The surface tension between generalized cells determines whether they can invade the surrounding stromal tissue. For *P*_*m*_ = 0.1,0.2 and *δ*_*am*_ = 0.5, the average surface tension stays positive at all times (see Fig 13). For all other combinations of *P*_*m*_ and *δ*_*am*_, the average surface tension eventually decreases below zero, after which the number of clusters rapidly increases. The number of Necrotic generalized cells decreases a short time later. These observations show that, even in the absence of other factors stimulating invasiveness, a high rate of cadherin mutation enables rapid metastasis. Consequently, studies of the regulation of adhesion molecules may give researchers clues to designing therapies to slow the rate of increase of tumor invasiveness. Such therapies would be especially useful to patients undergoing any of the many treatments, which can increase the invasiveness of surviving tumor tissue.

### Average Lifetime and Total Travel Distance of Generalized Cells

**Fig 14:**
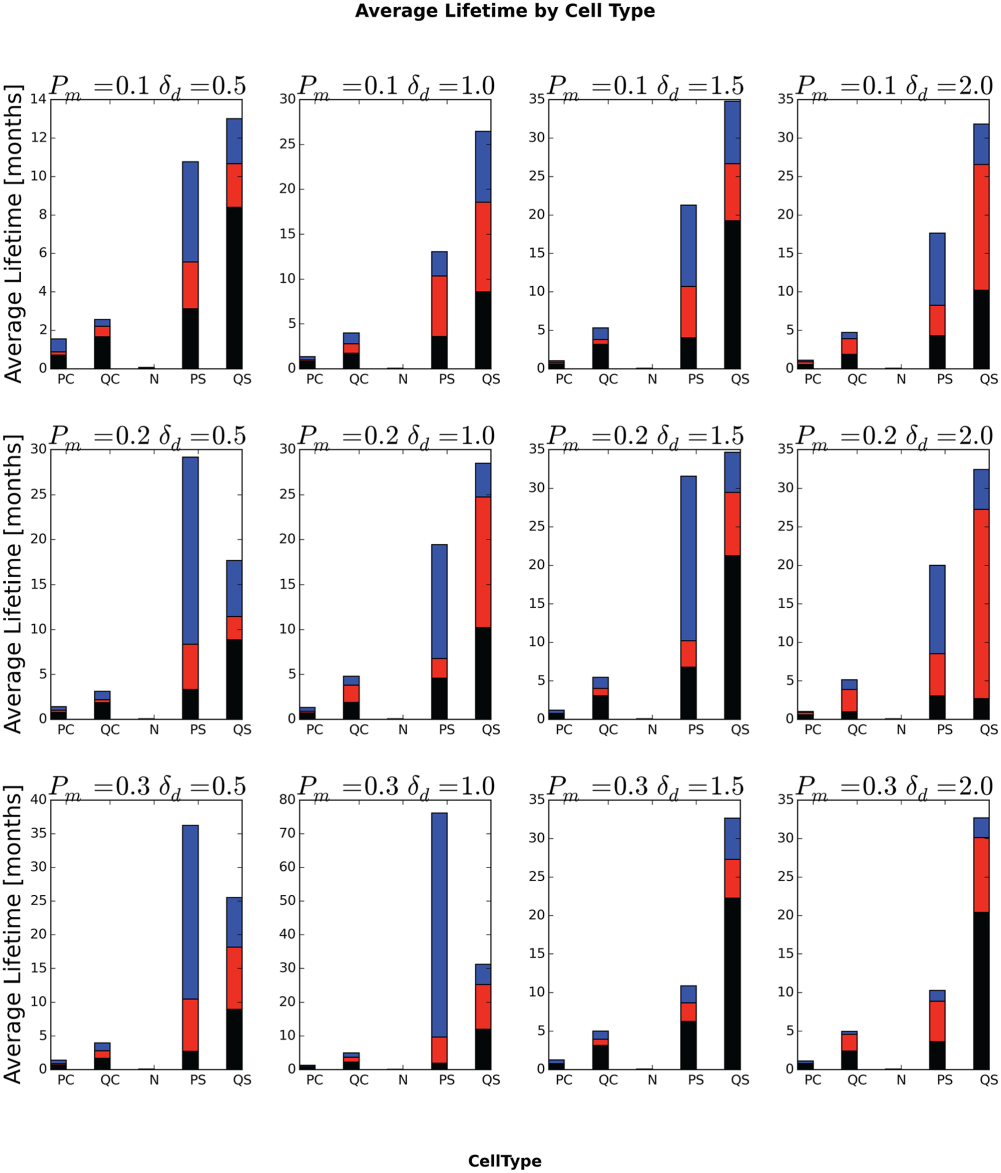
Average lifetime of generalized cells, by ***initial*** generalized-cell type. In each bar, the black-red boundary is the minimum generalized-cell lifetime, the red-blue boundary is the median generalized-cell lifetime and the top of the blue portion is the maximum generalized-cell lifetime, all averaged over all cells of the specified ***initial*** generalized-cell type and over all replicas for a given *P*_*m*_ and *δ*_*am*_.

**Fig 15:**
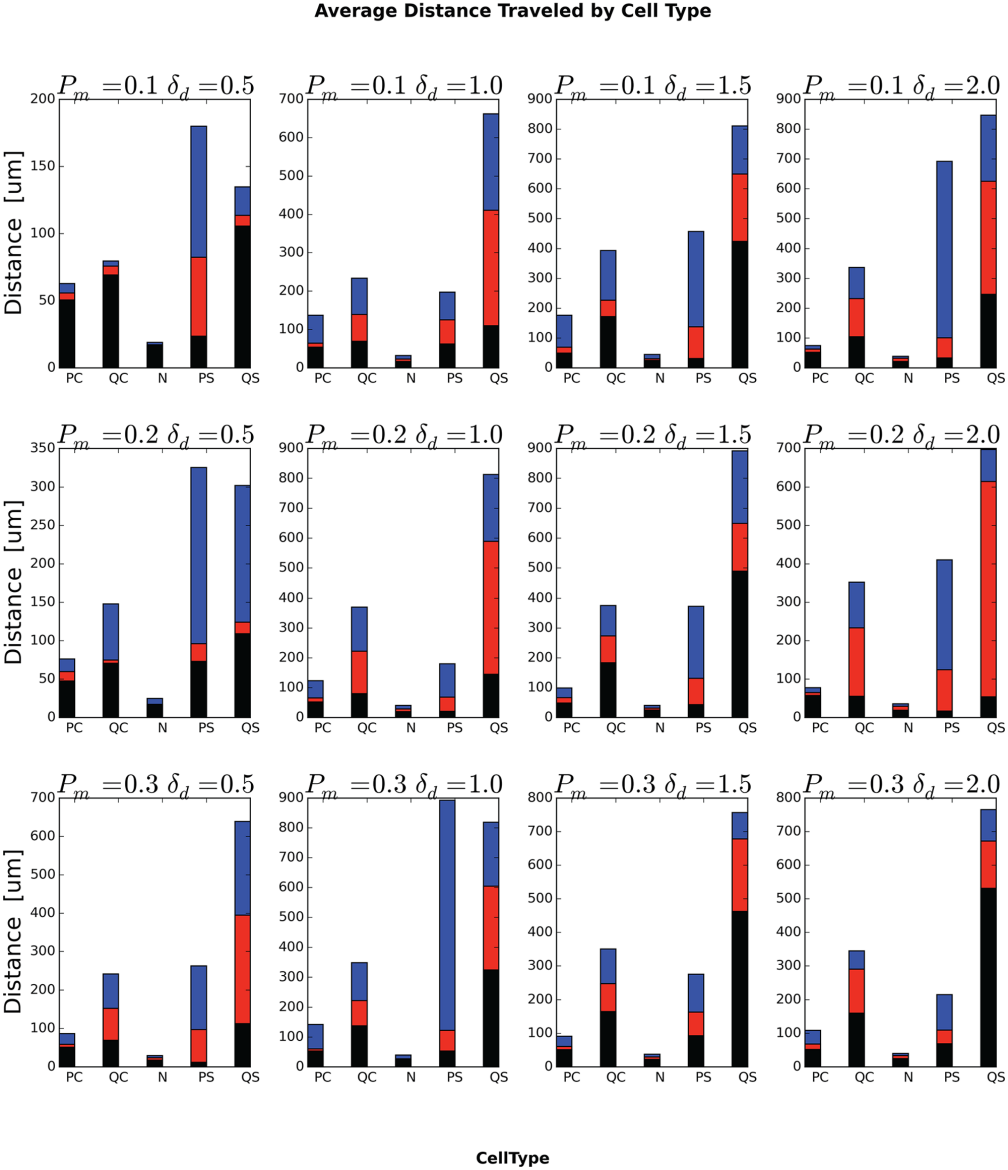
Average total distance traveled by generalized cells, by ***initial*** generalized-cell type. In each bar, the black-red boundary is the minimum total distance, the red-blue boundary is the median total distance and the top of the blue portion is the maximum total distance, all averaged over all cells of the specified ***initial*** generalized-cell type and over all replicas for a given *P*_*m*_ and *δ*_*am*_.

Fig 14 shows the lifetimes for different initial generalized-cell types for different values of *P*_*m*_ and *δ*_*am*_. As we expect, Necrotic generalized cells have short lifetimes and total travel distances, so the period a generalized cell spends in necrosis has a small effect on its total lifetime and travel distance. Quiescent non-stem generalized cells (QC) clearly have a lower probability of dying by senescence than proliferating non-stem generalized cells, since they do not divide, but, since quiescent generalized cells are closer to glucose-depleted regions than proliferating generalized cells, we might expect them also to have a higher probability of entering a glucose-depleted region and becoming necrotic, shortening their lifetime. However, in all cases, the proliferating non-stem-like generalized cells have a shorter lifetime than the quiescent non-stem-like generalized cells, indicating that necrosis is more important than starvation in determining their lifetime. As a result, initially quiescent non-stem-like generalized cells travel a longer total distance than initially proliferating non-stem-like generalized cells (Fig 15). Since stem-like generalized cells do not die from senescence, but are otherwise identical in properties to non-stem-like generalized cells, we would expect them to live longer than non-stem-like generalized cells, and, indeed, the typical lifetime of stem-like generalized cells is several-fold greater than that of non-stem-like generalized cells. As a result, initially proliferating stem-like generalized cells travel a longer total distance than proliferating non-stem-like generalized cells and quiescent stem-like generalized cells travel a longer total distance than quiescent non-stem-like generalized cells (Fig 15). The relationship between proliferating and quiescent stem-like generalized cells is more complex. Since these cells generalized do not experience senescence we would expect that proliferating stem-like generalized cells would have longer lifetimes than quiescent stem-like generalized cells. We see this relationship only for *P*_*m*_ = 0.2, *δ*_*am*_ = 0.5, *P*_*m*_ = 0.3, *δ*_*am*_ = 0.5 and *P*_*m*_ = 0.3 and *δ*_*am*_ = 1.0. In addition, for stem-like generalized cells, the relative lifetimes do not strictly correspond to the total travel distances, with the longer-lived type having a shorter total travel distance for *P*_*m*_ = 0.1, *δ*_*am*_ = 0.5, *P*_*m*_ = 0.2, *δ*_*am*_ = 0.5, *P*_*m*_ = 0.3, *δ*_*am*_ = 0.5.

## Conclusion

As in clinical and experimental cancer progression, simulation replicas of our model with the same parameters can produce a range of outcomes. However, for all replicas in the parameter range we studied, the qualitative evolution of adhesivity and initiation of metastasis agree with experimental observations of cell-behavior evolution in tumors, with single generalized cells or clusters of generalized cells developing high motility and detaching from the primary tumor [19], showing that the evolutionary trend and its consequences are robust. Together, these results suggest that weakening cell-cell adhesion and strengthening cell-stromal-tissue adhesion are primary enablers of metastasis.

### Future Work

Our simple model provides an easy-to-extend framework for exploring additional determinants of tumor evolution, some of which are difficult or impossible to measure or control *in vitro* and *in vivo*. The model enables tracking of cell-scale population heterogeneity and dynamics, cell-lineages, fitness and selection pressures. Most importantly, it also allows prediction of population-level drift (rates of evolution) of cell behaviors as a function of a limited number of experimentally-accessible parameters. These predicted population-level drifts can identify the factors most likely to promote or retard cancer progression and thus optimize cancer therapies. Thus extensions of our model can provide deeper insight into what drives tumor evolution, especially when we couple signaling and metabolic network models with cell- and tissue-behavior models. This coupling bridges the gap between molecular-scale observations and tissue-scale tumor progression. *E.g.*, in real tumors, changes in the adhesive properties of cells result from both heritable changes in the form or number of cell adhesion molecules and from cells’ response to hypoxia in tumors [28]. We are conducting simulations to explore the differences in tissue-level phenotype of these two mechanisms, alone and in combination. We are also simulating the effect on tumor phenotypes of variations in the fraction of stem-like progeny of stem-like-cell divisions, which prior computational studies have suggested promote increased agressiveness in surviving tumor tissue after therapy [21]. Adding a model of ECM alignment due to tension forces created by the tumor cells would be another natural extension of our model, since in *in vitro* experiments, cells follow aligned ECM fibers when they leave the primary tumor [19].

## Appendix: Simulation Implementation

This appendix presents the CC3D simulation implementation of the biomodels from the main text. The listings are in CC3DML and Python.

### Generalized-Cell Type Specification

The simulation defines six generalized-cell types (Medium, proliferating non-stem (PCancer), quiescent non-stem (QCancer), Necrotic, proliferating stem (PStem) and quiescent stem (QStem) generalized cells, which we abbreviate as M, PC, QS, N, PS, QS in the text) (see subsection “Cells” under “Biological Components”). Table 2 defines these generalized cell types in CC3DML:

**Table 2:** Generalized-cell type definitions in CC3DML.

<Plugin Name=“CellType”> <CellType TypeName=“Medium”TypeId=“0”/> <CellType TypeName=“PCancer”TypeId=“1”/> <CellType TypeName=“QCancer”TypeId=“2”/> <CellType TypeName=“Necrotic”TypeId=“3”/> <CellType TypeName=“PStem”TypeId=“4”/> <CellType TypeName=“QStem”TypeId=“5”/> </Plugin>

### Generalized-Cell Adhesion

Table 3 defines the generalized cells’ type-dependent initial molecular densities of cadherin and integrin adhesion molecules:

**Table 3:** Initial adhesion molecule density definition in CC3DML.

<Plugin Name=“AdhesionFlex”> <AdhesionMolecule Molecule=“Cad”/> <AdhesionMolecule Molecule=“Int”/> <AdhesionMolecule Molecule=“FN”/> <AdhesionMoleculeDensity CellType=“PCancer”Molecule=“Cad”Density=“8.0”/> <AdhesionMoleculeDensity CellType=“PCancer”Molecule=“Int”Density=“8.0”/> <AdhesionMoleculeDensity CellType=“PCancer”Molecule=“FN”Density=“0”/> <AdhesionMoleculeDensity CellType=“QCancer”Molecule=“Cad”Density=“8.0”/> <AdhesionMoleculeDensity CellType=“QCancer”Molecule=“Int”Density=“8.0”/> <AdhesionMoleculeDensity CellType=“QCancer”Molecule=“FN”Density=“0”/> <AdhesionMoleculeDensity CellType=“NCancer”Molecule=“Cad”Density=“8.0”/> <AdhesionMoleculeDensity CellType=“Necrotic”Molecule=“Int”Density=“8.0”/> <AdhesionMoleculeDensity CellType=“Necrotic”Molecule=“FN”Density=“0”/> <AdhesionMoleculeDensity CellType=“Medium”Molecule=“FN”Density=“16.0”/> <AdhesionMoleculeDensity CellType=“PStem”Molecule=“Cad”Density=“8.0”/> <AdhesionMoleculeDensity CellType=“PStem”Molecule=“Int”Density=“8.0”/> <AdhesionMoleculeDensity CellType=“PStem”Molecule=“FN”Density=“0”/> <AdhesionMoleculeDensity CellType=“QStem”Molecule=“Cad”Density=“8.0”/> <AdhesionMoleculeDensity CellType=“QStem”Molecule=“Int”Density=“8.0”/> <AdhesionMoleculeDensity CellType=“QStem”Molecule=“FN”Density=“0”/> <BindingFormula Name=“Binary”> <Formula>min(Molecule1, Molecule2)</Formula> <Variables> <AdhesionInteractionMatrix> <BindingParameter Molecule1=“Cad”Molecule2=“Cad”>2.0</BindingParameter> <BindingParameter Molecule1=“Int”Molecule2=“FN”>0.2</BindingParameter> </AdhesionInteractionMatrix> </Variables> </BindingFormula> <NeighborOrder>3</NeighborOrder> </Plugin>

We first define the internal adhesion-molecule names Cadherin (Cad), Integrin (Int), and Fibronectin (FN). The AdhesionMoleculeDensity tags describe the initial concentration of adhesion molecules at the surface of generalized cells of the type specified as a CellType attribute. In the <BindingFormula> section we specify the mathematical formula for $F\left(N_{m}^{i},N_{n}^{j}\right)$—see Eq (7)—as:

<Formula>min(Molecule1, Molecule2)</Formula>

In the <AdhesionInteractionMatrix> section we specify *k*_*m*, *n*_, the affinity coefficients between different adhesion molecule classes. Unspecified combinations of adhesion classes have their affinity coefficient set to 0. Finally, we set *N*_max_ in Eq (9) using the <NeighborOrder> tag.

### Glucose Transport

Since Glucose diffusion is fast compared to the rate of cell reorganization, Glucose reaches its steady-state distribution. Consequently we set the left-hand side of Eq (15) to zero and use the following CC3DML syntax for the CC3D steady-state diffusion solver:

**Table 4:** Steady state diffusion solver expressed in CC3DML

<Steppable Type=“SteadyStateDiffusionSolver2D”Frequency=“10”> <DiffusionField> <DiffusionData> <FieldName>Glucose</FieldName> <DiffusionConstant>13500.0</DiffusionConstant> <DecayConstant>0.45</DecayConstant> </DiffusionData> <SecretionData> <Uptake Type=“QStem” MaxUptake=“1.69”MichaelisMentenCoef=“0.00256”/> <Uptake Type=“PStem” MaxUptake=“2.25”MichaelisMentenCoef=“0.00256”/> <Uptake Type=“QCancer” MaxUptake=“1.69”MichaelisMentenCoef=“0.00256”/> <Uptake Type=“PCancer” MaxUptake=“2.25”MichaelisMentenCoef=“0.00256”/> <Secretion Type=“Medium”>0.145</Secretion> </SecretionData> </DiffusionField> </Steppable>

### Generalized Cell Health and Damage Algorithm

In the StarvationDamageAcumulator Python steppable we define *M* (*x*), see Eq (18) as:

**Table 5:** Steady state diffusion solver expressed in CC3DML

def MM(self, x, m, k): return (m*x/(x + k))

For PCancer, QCancer, PStem, QStem cells, we increment their starvation and health factors using the Python code as follows:

**Table 6:** Python implementation of starvation and health factor calculations.

**for** cell **in** self.cellList: **if** cell.**type**!= self.NECROTIC: cellDict = CompuCell.getPyAttrib(cell) pt = getCellCOMPoint3D(cell) conc = glucoseField.get(pt) **if** cell.**type** == self.PCANCER: **if** conc < ip.GluD: cellDict[“Starv”]+=**abs**(self.MM(conc, ip.PUgMax, ip.GluK)\ - self.MM(ip.GluD, ip.PUgMax, ip.GluK)) **else**: cellDict[“Health”]+=self.MM(conc, ip.PUgMax, ip.GluK)\ - self.MM(ip.GluD, ip.PUgMax, ip.GluK) **if** cell.**type** == self.QCANCER: **if** conc < ip.GluD: cellDict[“Starv”]+=abs(self.MM(conc, ip.QUgMax, ip.GluK)\ - self.MM(ip.GluD, ip.QUgMax, ip.GluK)) **else**: cellDict[“Health”]+=self.MM(conc, ip.QUgMax, ip.GluK)\ - self.MM(ip.GluD, ip.QUgMax, ip.GluK) **if** cell.**type** == self.PSTEM: **if** conc < ip.GluD: cellDict[“Starv”]+=**abs**(self.MM(conc, ip.SUgMax, ip.GluK)\ **else**: cellDict[“Health”]+=self.MM(conc, ip.SUgMax, ip.GluK)\ - self.MM(ip.GluD, ip.SUgMax, ip.GluK) **if** cell.**type** == self.QSTEM: **if** conc < ip.GluD: cellDict[“Starv”]+=**abs**(self.MM(conc, ip.QSUgMax, ip.GluK)\ - self.MM(ip.GluD, ip.QSUgMax, ip.GluK)) **else**: cellDict[“Health”]+=self.MM(conc, ip.QSUgMax, ip.GluK)\\ - self.MM(ip.GluD, ip.QSUgMax, ip.GluK)

The ip.GluD variable stores the *x*_*thresh*_ value for the critical glucose concentration for PCancer cells. ip.PUgMax is the maximum uptake rate of glucose (*m* in Eq (18)) and ip.GluK is the constant *k* in Eq (18). Similarly ip.QUgMax,ip.SUgMax and ip.QSUgMax are the maximum uptake rates for QCancer, PStem and QStem respectively.

### Generalized Cell Type Transition Algorithm

We specify the algorithm to implement generalized-cell type changes as follows:

**Table 7:** Python implementation of cell-growth algorithm.

**for** cell **in** self.cellList: cellDict = CompuCell.getPyAttrib(cell) **if** cell.**type** == self.PCANCER: **if** cellDict[“Starv”] > ip.PNeThr0: cell.**type** = self.NECROTIC cellDict[“Health”]=0 **if** cell.type==self.QCANCER: **if** cellDict[“Starv”] > ip.QNeThr0: cell.type = self.NECROTIC cellDict[“Health”]=0 **if** cellDict[“Health”] > ip.QPThr0: cell.**type** = self.PCANCER cellDict[“Health”]=0 **if** cell.**type** == self.PSTEM: if cellDict[“Starv”] > ip.SNeThr0: cell.**type** = self.NECROTIC cellDict[“Health”]=0 **if** cell.**type** == self.QSTEM: if cellDict[“Starv”] > ip.QSNeThr0: cell.**type** = self.NECROTIC cellDict[“Health”]=0 **if** cellDict[“Health”] > ip.QSSThr0: cell.**type** = self.PSTEM cellDict[“Health”]=0

### Generalized-Cell Growth and Death Algorithm

The generalized-cell growth and death algorithm can be conveniently expressed in Python code as follows:

**Table 8:** Python implementation of generalized-cell growth and death.

**for** cell **in** self.cellList: pt = getCellCOMPoint3D(cell) conc = glucoseField.get(pt) cellDict = CompuCell.getPyAttrib(cell) **if** cell.**type** == self.NECROTIC: cell.targetVolume-=**min**(ip.decvol, cell.targetVolume) cell.targetSurface = ip.ktgs*sqrt(cell.targetVolume) **if** cell.**type** == self.PCANCER: cell.targetVolume+=ip.incvol*max(0, conc—ip.PGrThr0) cell.targetSurface = ip.ktgs*sqrt(cell.targetVolume) **if** cell.**type** == self.PSTEM: cell.targetVolume+=ip.incvol*max(0, conc—ip.SGrThr0) cell.targetSurface = ip.ktgs*sqrt(cell.targetVolume)

After each MCS, we iterate over all generalized cells in the simulation and shrink Necrotic generalized cells by a constant amount (ip.decvol) provided that their volume is greater than their current target volume (cell.targetVolume). PCancer and PStem generalized cells grow according to Eq (21) where ip.PGrThr0 and ip.SGrThr0 store values of *G*_*thresh*_ from Eq (21) for PCancer and PStem generalized cells respectively and ip.incvol stores the value of *k* from Eq (21). ip.ktgs stores the value for *q*_*s*.*v*_ (see Eq (23)).

### Mitosis, Aging and Mutation Algorithms

**Table 9:** Python code selecting generalized cells which will undergo mitosis.

**def** step(self, mcs): ip = self.parameters cells_to_divide=[] **for** cell **in** self.cellList: **if** ((cell.**type**==self.PCANCER **or** cell.**type**==self.QCANCER)\ **and** cell.volume > ip.volmaxmit)\ **or** ((cell.**type**==self.PSTEM **or** cell.**type**==self.QSTEM)\ **and** cell.volume > ip.Svolmaxmit): cells_to_divide.append(cell) **for** cell **in** cells_to_divide: self.divideCellRandomOrientation(cell)

ip.volmaxmit stores the value of the doubling volume for PCancer and QCancer generalized cells whereas ip.Svolmaxmit stores the doubling volume for PStem and QStem generalized cells. The first for loop builds a list of generalized cells to divide and division takes place in the second for loop. In our model quiescent generalized cells do not grow however, a growing proliferating generalized cell with a volume close to doubling volume can become quiescent. In this special situation quiescent generalized cell can divide.

**Table 10:** Python implementation (partial) of the updateAttributes function which modifies the properties of parent and daughter generalized cells after mitosis.

**def** updateAttributes(self): parentCell = self.mitosisSteppable.parentCell childCell = self.mitosisSteppable.childCell parentCell.targetVolume = ip.V0 childCell.targetVolume = ip.V0 parentCell.targetSurface = ip.ktgs*sqrt(parentCell.targetVolume) childCell.targetSurface = ip.ktgs*sqrt(childCell.targetVolume) parentCell.lambdaVolume = ip.LBD_V0 parentCell.lambdaSurface = ip.LBD_S0 childCell.lambdaVolume = ip.LBD_V0 childCell.lambdaSurface = ip.LBD_S0 parentCellDict = CompuCell.getPyAttrib(parentCell) childCellDict = CompuCell.getPyAttrib(childCell) **if** parentCell.**type** == self.PCANCER: temp = random.gauss(ip.maxdiv,2) **if** parentCellDict[“Counter”] <= temp: parentCell.**type** = self.QCANCER # both cells are QC after mitosis childCell.**type** = self.QCANCER parentCellDict[“Counter”]+=1 childCellDict[“Counter”]=deepcopy(parentCellDict[“Counter”]) **if** parentCellDict[“Counter”] > temp: parentCell.**type** = self.NECROTIC childCell.**type** = self.NECROTIC **if** parentCell.**type** == self.PSTEM or parentCell.type == self.QSTEM:# Stem cell parentCellDict[“Counter”]+=1 parentCell.**type** = self.QSTEM *# One is QS and* childCell.**type** = self.QCANCER *# the other is QC* **if** random.random() <= ip.probstem: *# 0.2 chance for a 2nd QS* childCell.**type** = self.QSTEM childCellDict[“Counter”]=0 parentCellDict[“Starv”]=0 childCellDict[“Starv”]=0 parentCellDict[“Health”]=0 childCellDict[“Health”]=0

**Table 11:** Python implementation of updateAttributes function (continuation of Table 10) to randomly mutate cadherin expression levels.

#*getting current Cad and Int values* jcadh = self.adhesionFlexPlugin.getAdhesionMoleculeDensityByIndex(parentCell,0) jint = self.adhesionFlexPlugin.getAdhesionMoleculeDensityByIndex(parentCell,1) jFN = self.adhesionFlexPlugin.getAdhesionMoleculeDensityByIndex(parentCell,2) #*probability for mutation* **if** parentCell.**type**!= self.NECROTIC: r = random.random() **if** r < ip.probmut: new_cadh = random.gauss(jcadh, ip.cadhstdev) **if** new_cadh >= 0 **and** new_cadh <= 16: jcadh = new_cadh r = random.random() **if** r < ip.probmut: new_int = random.gauss(jint, ip.cadhstdev) **if** new_int >= 0 **and** new_int <= 16: jint = new_int *# setting new adhesion density for product cell* self.adhesionFlexPlugin.assignNewAdhesionMoleculeDensityVector(parentCell, \ [jcadh, jint, jFN]) self.adhesionFlexPlugin.assignNewAdhesionMoleculeDensityVector(childCell, \ [jcadh, jint, jFN])

### Helper CompuCell3D modules, initial conditions and basic GGH parameters

Here we present the CC3DML modules which track centers of mass and monitor sets of pixels belonging to each individual generalized cell:

**Table 12:** CC3DML instantiation of center of mass and pixel tracker modules.

<Plugin Name=“CenterOfMass”/> <Plugin Name=“PixelTracker”/>

As an initial condition, we single 3x3 voxel quiescent stem cell located in the middle of the lattice:

**Table 13:** CC3DML specification of initial generalized cell layout.

<Steppable Type=“UniformInitializer”> <Region> <BoxMin x=“250”y=“250”z=“0”> <BoxMax x=“253”y=“253”z=“1”> <Width>3</Width> <Gap>0</Gap> <Types>QStem</Types> </Region> </Steppable>

Finally, we specify thelattice dimensions (<Dimensions>), boundary conditions (<Boundary_x>,<Boundary_y>), average amplitude of generalized-cell membrane fluctuation (<Temperature>), neighbor range used to pick source and target voxels for the voxel copy attempt (<NeighborOrder>), duration of the simulation in MCS (<Steps>) and the seed for the random number generator (<RandomSeed>) in CC3DML as follows:

**Table 14:** CC3DML specification of basic simulation properties.

<Potts> <Dimensions x=“500”y=“500”z=“1”/> <Steps>1000000</Steps> <Temperature>50</Temperature> <RandomSeed>2284322</RandomSeed> <NeighborOrder>3</NeighborOrder> <Boundary_x>Periodic</Boundary_x> <Boundary_y>Periodic</Boundary_y> </Potts>

### Simulation parameters

For completeness, Table 1 lists the parameters that generated Fig 2.
